# Genome amplification and cellular senescence are hallmarks of human placenta development

**DOI:** 10.1371/journal.pgen.1007698

**Published:** 2018-10-12

**Authors:** Philipp Velicky, Gudrun Meinhardt, Kerstin Plessl, Sigrid Vondra, Tamara Weiss, Peter Haslinger, Thomas Lendl, Karin Aumayr, Mario Mairhofer, Xiaowei Zhu, Birgit Schütz, Roberta L. Hannibal, Robert Lindau, Beatrix Weil, Jan Ernerudh, Jürgen Neesen, Gerda Egger, Mario Mikula, Clemens Röhrl, Alexander E. Urban, Julie Baker, Martin Knöfler, Jürgen Pollheimer

**Affiliations:** 1 Department of Obstetrics and Gynaecology, Reproductive Biology Unit, Medical University of Vienna, Vienna, Austria; 2 Children's Cancer Research Institute, St. Anna Children´s Hospital, Vienna, Austria; 3 Biooptics Facility of Institute of Molecular Pathology, Institute of Molecular Biotechnology and Gregor Mendel Institute, Vienna, Austria; 4 Department of Gynecological Endocrinology and Reproductive Medicine, Medical University of Vienna, Vienna, Austria; 5 Department of Psychiatry and Behavioral Sciences, Stanford University, Stanford, California, United States of America; 6 Center for Pathobiochemistry and Genetics, Medical University of Vienna, Vienna, Austria; 7 Department of Genetics, Stanford University School of Medicine, Stanford, California, United States of America; 8 Department of Clinical and Experimental Medicine, Linköping University, Linköping, Sweden; 9 Department of Clinical Immunology and Transfusion Medicine, and Department of Clinical and Experimental Medicine, Linköping University, Linköping, Sweden; 10 Clinical Institute of Pathology, Medical University of Vienna, Vienna, Austria; 11 Department of Psychiatry and Behavioral Sciences, Department of Genetics, Stanford University School of Medicine, Tasha and John Morgridge Faculty Scholar, Stanford Child Health Research Institute, Stanford, California, United States of America; Max Planck Institute for Molecular Genetics, GERMANY

## Abstract

Genome amplification and cellular senescence are commonly associated with pathological processes. While physiological roles for polyploidization and senescence have been described in mouse development, controversy exists over their significance in humans. Here, we describe tetraploidization and senescence as phenomena of normal human placenta development. During pregnancy, placental extravillous trophoblasts (EVTs) invade the pregnant endometrium, termed decidua, to establish an adapted microenvironment required for the developing embryo. This process is critically dependent on continuous cell proliferation and differentiation, which is thought to follow the classical model of cell cycle arrest prior to terminal differentiation. Strikingly, flow cytometry and DNAseq revealed that EVT formation is accompanied with a genome-wide polyploidization, independent of mitotic cycles. DNA replication in these cells was analysed by a fluorescent cell-cycle indicator reporter system, cell cycle marker expression and EdU incorporation. Upon invasion into the decidua, EVTs widely lose their replicative potential and enter a senescent state characterized by high senescence-associated (SA) β-galactosidase activity, induction of a SA secretory phenotype as well as typical metabolic alterations. Furthermore, we show that the shift from endocycle-dependent genome amplification to growth arrest is disturbed in androgenic complete hydatidiform moles (CHM), a hyperplastic pregnancy disorder associated with increased risk of developing choriocarinoma. Senescence is decreased in CHM-EVTs, accompanied by exacerbated endoreduplication and hyperploidy. We propose induction of cellular senescence as a ploidy-limiting mechanism during normal human placentation and unravel a link between excessive polyploidization and reduced senescence in CHM.

## Author summary

In tissues, cellular differentiation is normally associated with cell cycle arrest. However, in some cases differentiating cells continue their cyclic activity in the absence of cell division. This event, referred to as endoreduplication, leads to polyploidy, which means an increased number of chromosome sets per cell and has been demonstrated in rodent placental trophoblast giant cells (TGCs). However, it is unknown whether human placental trophoblasts also endoreduplicate their genome. To study this, we focused on extravillous trophoblasts (EVTs), a specific trophoblastic subtype that invades the uterus during pregnancy in order to control blood supply and nutrient transfer to the growing embryo. We show that initiation of EVT differentiation is characterized by induced endoreduplication leading to genomic tetraploidization. Different to TGCs, EVTs duplicate their genome in an even manner. Upon invasion into the uterus, EVTs stop their endoreduplicative cycle and undergo cellular senescence. We further show that EVTs of hyperplastic complete hydatidiform mole (CHM) placentas, a genetic pregnancy disorder, are characterized by exacerbated endoreduplication, a greater DNA content and reduced signs for senescence. In summary, we propose senescence as a ploidy limiting factor during placental development and describe its suppression in hyperploid CHM-EVTs.

## Introduction

It is commonly assumed that trophoblasts of the human placenta exit their cell cycle as they develop into differentiated subtypes. This hypothesis follows the assumption that quiescence is a consequence of terminal differentiation in tissues. In detail, the villous epithelium of the human placenta hosts progenitor cells that either fuse to form multinucleated, hormone-secreting syncytiotrophoblasts or differentiate into invasive extravillous trophoblasts (EVTs). In placental anchoring villi, the latter undergo a multi-step differentiation process that starts at the epidermal growth factor-positive (EGFR^+^) proximal cell column (CC), characterized by high proliferative activity. These cells further differentiate into non-dividing human leukocyte antigen G-positive (HLA-G^+^) distal CC trophoblasts and invade the endometrial epithelium of the pregnant uterus, termed decidua. Remarkably, EVTs fulfil a great variety of functions including vascular remodelling [1, 2], interaction with immune cells [3, 4] as well as defense against pathogens [5] and they undergo an epithelial to mesenchymal-like transition [6]. In rodents, invasive trophoblastic giant cells (TGCs), the functional equivalent to human EVTs, contain a highly polyploid DNA content [7, 8]. Differentiation of mouse TGCs is characterized by the omission of mitosis and induction of endoreduplication by undergoing multiple rounds of S- and G-phases [9]. A single polyploid TGC nucleus contains up to 1000 copies of the genome [10]. Functional studies showed that oscillating levels of KIP2p57 (from now on referred to as p57) in G-phase as well as Cyclin E and A in S-phase are necessary for DNA polyploidization in rodent TGCs [11, 12]. Well in line, genomic deletion of Cyclin E or p57 prevents trophoblast endoreduplication in mice [13–15]. While it was assumed for years that polyploidization in TGCs results in a linear amplification of the entire genome, recent data showed that endoreduplication in TGCs leads to under- and overreplication of specific genomic regions [16, 17]. In contrast to rodents, our knowledge about endoreduplication in human trophoblasts is scarce. For instance, very little data exist to suggest an increased DNA content in human EVTs [18–20]. Moreover, it is not clear at which stage of differentiation the proposed increase in DNA copy numbers occurs as the currently accepted model suggests that generation of trophoblasts, expressing the prime EVT marker HLA-G, is accompanied with terminal growth arrest of these cells [21]. In addition, whether DNA amplification in human EVTs affects the entire genome or is characterized by copy number variations (CNVs) of specific chromosomes and/or genes is currently unknown. As mentioned above, different to rodent invasive trophoblasts, HLA-G^+^ trophoblasts are thought to enter a growth arrested status prior to terminal differentiation. Commonly, end-differentiated cells in tissues undergo cellular quiescence by entering G0 [22]. In vitro, entry into the G0-phase can be provoked by nutrient starvation, cell contact inhibition or signals that induce differentiation [22]. Cellular quiescence is reversible as noticed for instance in endothelial cells during wound healing and angiogenesis or in activated stem cells [23]. In addition, G0-phase cells are usually defined as metabolically and transcriptionally less active than dividing or senescent cells reflecting an end-point differentiational program which dictates cells to execute highly specific functions [24]. In contrast to quiescence or G0, senescent cells are in an irreversible growth arrest [25]. This status is believed to be induced by various stimuli of which telomeric shortening and stress are the best characterized triggers [22]. Although senescent cells do not differ from quiescent cells by their DNA content, the former population shows an array of specific markers or phenotypical characteristics. The most commonly used markers to identify senescent cells include beta-galactosidase (βG) activity [26] together with the induction of cyclin-dependent kinase inhibitors (CDKNs) such as p16 [27], p21 [28] and p57 [29, 30]. Senescence is accompanied by high transcriptional and metabolic activity [31] as well as induction of a so-called senesecence-associated secretory phenotype (SASP) [32]. In addition, when cells senesce they often increase in size, show a tetraploid phenotype and, different to quiescent cells, remain in a G1-arrest [24]. Apart from its induction at the end of the cellular replicative lifespan, senescence is thought to be triggered by oncogenes, DNA damage and oxidative stress suggesting senescence as part of a pathological signature [24]. This view has recently been challenged by demonstrating that cellular senescence has its origin in embryonic development in mice and likely is involved in tissue remodelling [33, 34].

The aim of this study was to firstly characterize the cell cycle status and genomic amplification during the different stages of EVT differentiation. Secondly, we asked whether terminal differentiation in EVTs is associated with induction of senescence. Finally, we studied cases of complete hydatidiform moles (CHM) addressing the question whether the hyperplastic phenotype in CHM would affect ploidy, cell cycle and senescence in CHM-EVTs.

## Results

### EVTs undergo tetraploidization

**Fig 1:**
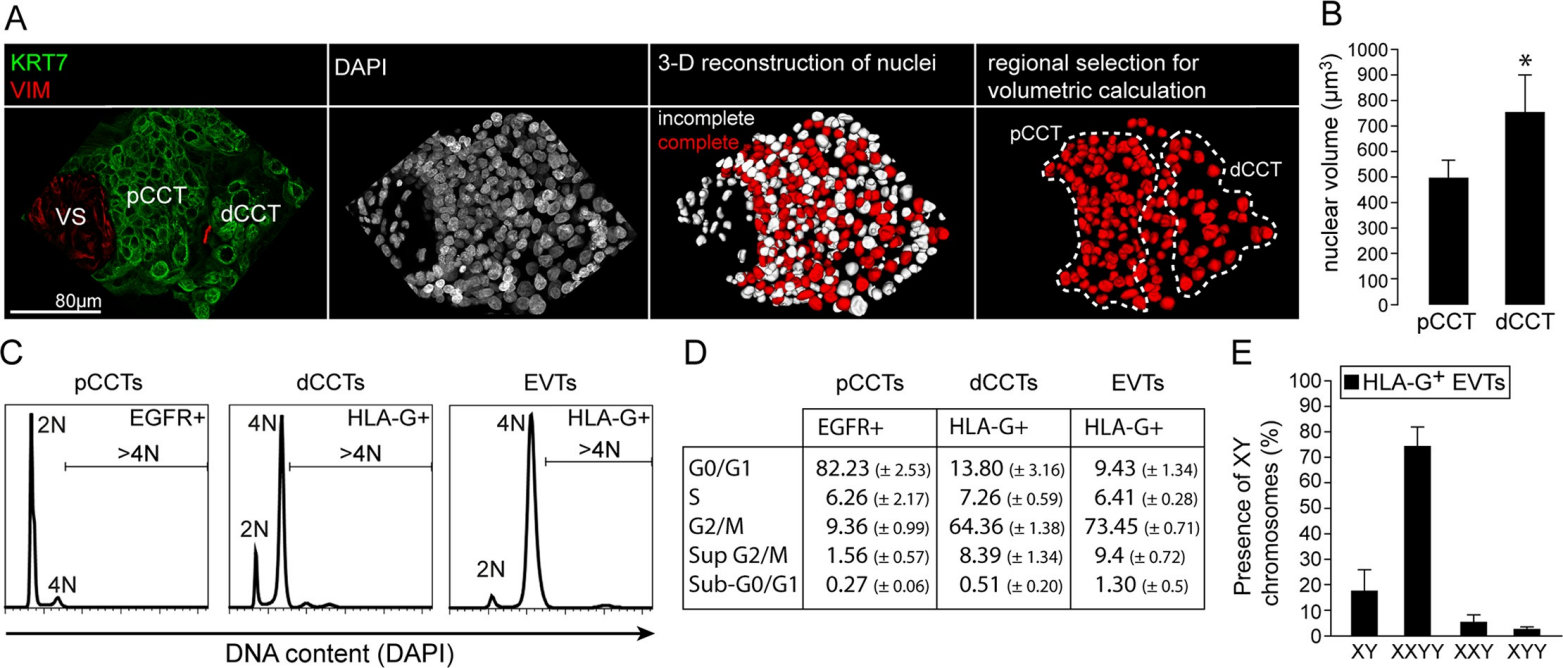
Polyploidization of human trophoblasts correlates with HLA-G expression. (A) Representative IF co-staining showing keratin7 (green) and vimentin (VIM, red), DAPI (grey) and a 3D reconstruction of DAPI signals of a placental villous tissue section (30μm) of the same image. Complete and incompletely reconstructed nuclei are shown in red and white, respectively. (B) Quantification of nuclear volumes obtained as demonstrated in A of proximal and distal cell column trophoblast nuclei (n = 5 cell columns of 3 placentas). (C) Flow cytometry analyses of DAPI signals representing DNA content of MACS-sorted EGFR+ and HLA-G+ CCTs or HLA-G-positive EVTs (n = 3). (D) Quantification and segmentation in cell cycle phases of data obtained from FC analyses (n = 3). (E) FISH analysis using probes against sex chromosomes X and Y of MACS-sorted HLA-G+ EVTs from decidua basalis tissue (n = 4 male placentas).

First we performed immune fluorescence (IF)-based phenotypical analyses of EGFR^+^ and HLA-G^+^ cell column trophoblasts. Interestingly, nuclei of HLA-G^+^ CCTs appear to be bigger than those of EGFR^+^ cells (S1A Fig). Magnetic bead-assisted isolation of these two populations revealed that HLA-G^+^ trophoblasts show a bigger nuclear diameter when compared to non-invasive EGFR^+^ trophoblasts (S1B and S1C Fig). To further study this phenomenon we performed Confocal Laser Scanning Microscopy (CLSM)-assisted measurement of nuclear volumes in 3-D reconstructed nuclei of cell column trophoblasts (Fig 1A). Again, these analyses revealed a significantly increased nuclear volume in distal CCTs when compared to proximal CCTs, suggesting a positive correlation between enhanced nuclear volume and EVT differentiation (Fig 1B). Moreover, flow cytometric (FC) analysis of DAPI content in EGFR^+^ and HLA-G^+^ CCTs as well as in HLA-G^+^ EVTs revealed that HLA-G^+^ trophoblasts show a predominant 4N status (Fig 1C). A small but significant subpopulation containing a DNA content beyond 4N was detectable in both HLA-G^+^ CCTs and EVTs (Fig 1D). In parallel, we isolated HLA-G^+^ EVTs from first trimester placental and deciudal tissues, which were pre-selected for an embryonic male 46, XY karyotype. Again, the majority of EVTs showed a tetraploid, mononuclear XXYY phenotype (Fig 1E). Figure S1D shows positive embryonic male and female nuclei and the graph in S1E Fig indicates the percentage of male and female cells after positive selection for HLA-G^+^ cells. Finally, we subjected EGFR^+^ and HLA-G^+^ CCTs to DNA deep sequencing (DNAseq). Significant CNVs were not found when comparing human polyploid HLA-G cells and diploid EGFR cells (S1F Fig, upper two plots). For comparison, mouse polyploid TGCs display a large amount of significant CNVs when compared to diploid embryonic cells (S1E Fig, lower plot). Altogether, these studies suggest a predominant tetraploid status in invasive, HLA-G^+^ human EVTs with no signs for CNVs.

### HLA-G^+^ trophoblasts are partly in an active cell cycle but show no signs for mitosis

**Fig 2:**
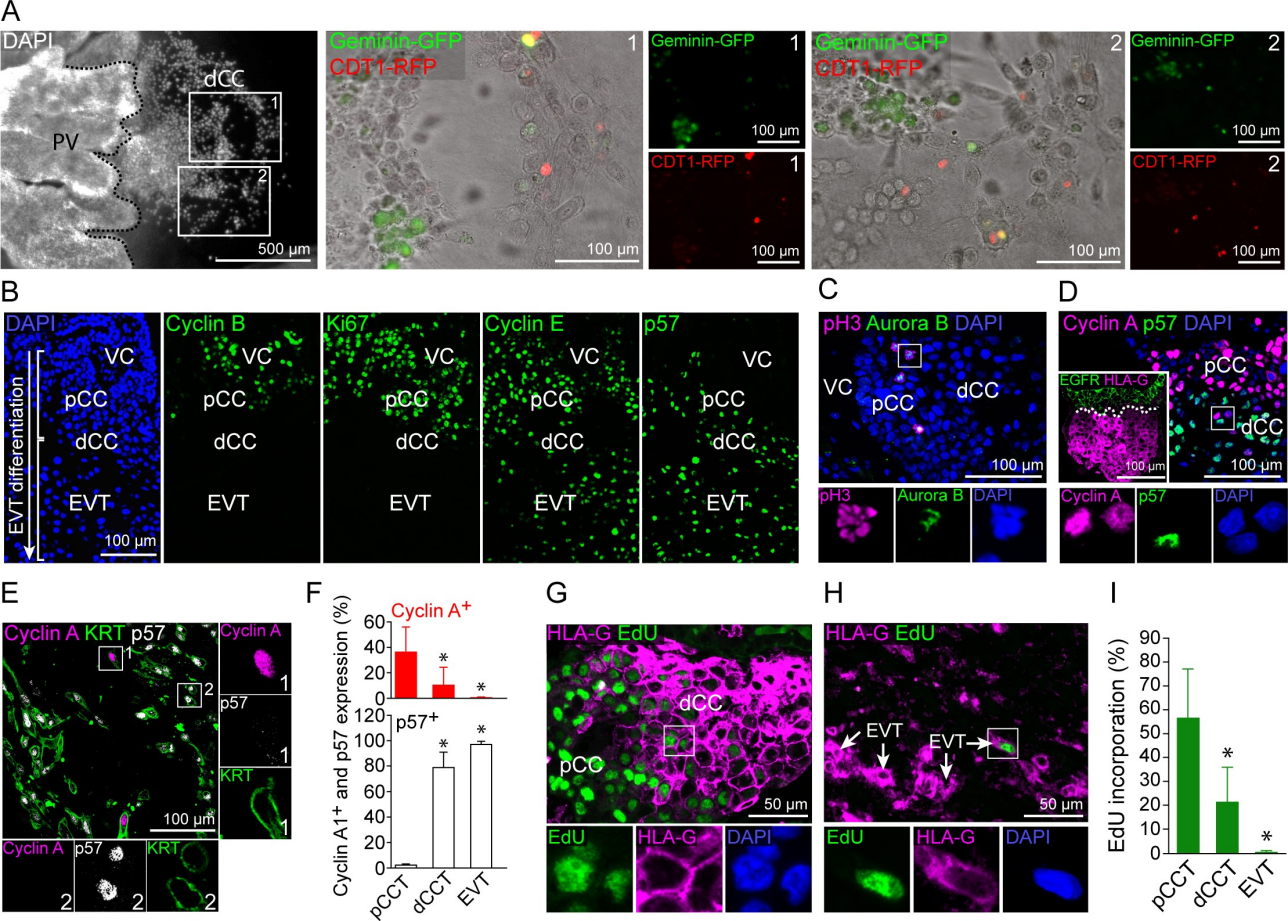
EVTs show cell cycle patterns of endoreduplication. (A) FUCCI cell cycle sensor analyses of outgrowing placental explants. Two different outgrowth-areas (boxes 1 and 2) of a representative placental explant (left picture) are shown in detail (n = 9 explants of 3 first trimester placentas). (B) IF stainings of serial tissue sections of a first trimester placental anchoring villous showing (from left to right) DAPI, Cyclin B, Ki67, Cyclin E and p57. (n = 3) (C) IF co-staining of first trimester placental tissue (n = 4) showing pH3 (magenta) and Aurora B (green) expressing mitotic figures (blue) in pCCTs. (D) IF co-staining of first trimester placental tissue (n = 4) showing Cyclin A (magenta) and p57 (green) in CCTs. EGFR (green) and HLA-G (magenta) double staining of a serial section is shown in the boxed insert (left bottom). (E) IF triple-staining of a first trimester decidua basalis tissue section (n = 5) showing Cyclin A (magenta), p57 (grey) and KRT7 (green) in EVTs. (F) Percentages of Cyclin A^+^ and p57^+^ in KRT^+^ pCCTs, dCCTs and EVTs (G) IF co-staining of first trimester tissue (n = 3) showing HLA-G (magenta) and EdU (green) incorporation into CCTs. (H) IF co-staining of first trimester tissue showing HLA-G (magenta) expression and EdU (green) incorporation into EVTs in decidua basalis tissue (n = 3). (I) Quantification of EdU incorporation into pCCT, dCCT and EVTs of cultivated first trimester tissue explants (n = 8). Digitally zoomed insets display a split-channel-depiction of the boxed area. DAPI was used to visualize nuclei.

Next, we addressed the question whether increased nuclear volume and DNA content observed in HLA-G^+^ trophoblasts correlate with an active endo-cycle in these cells. First, we transduced outgrowing first trimester placental explants with a BacMam FUCCI reporter system, labelling cells expressing Cdt1-RFP in G1/S phase (red) or geminin-GFP in G2/S/M phase (green). Cells in S phase express both reporters and thus appear in yellow. Interestingly, we found distal CCTs labelled in red, green or yellow indicative for an active cell cycle (Fig 2A). We also noticed some positive nuclei in detaching, migratory EVTs (S2A Fig). To further study this phenomenon, we determined the expression pattern of various cell cycle markers in first trimester placental anchoring villi harbouring both cell column trophoblasts and invading decidual EVTs. As expected pCCTs express high levels of Ki67 and mitosis-associated cyclin B, both of which were absent from invasive EVTs (Fig 2B). Interestingly, EVTs induce the endocycle-associated cell cycle regulators cyclin E and p57. Of note, p57 was the only CDKN tested with a clear expression profile in dCCTs and EVT whereas p21, p27 and p16 were generally absent or weakly expressed (S2D–S2F Fig). Further analyses revealed that mitosis-specific expression of phospho-histone H3 (pH3) and Aurora B are restricted to EGFR^+^ trophoblastic subpopulations including vCTBs (S2B Fig) and pCCTs (Fig 2C). A consecutive tissue section of Fig 2C showing localisation of pH3^+^/EGFR^+^ pCCs is presented in S2C Fig. Interestingly, HLA-G^+^ dCCTs and EVTs show reciprocal expression of the G1/S-phase cyclin A and p57 (Fig 2D and 2E). Double-positive HLA-G and cyclin A trophoblasts at the proximal and distal ends of the CC are shown in S2G Fig. In contrast, cyclin E^+^ nuclei of dCCTs and EVTs were also positive for p57, suggesting suppression of S-phase entry in these cells (S2H and S2I Fig). Evaluation of cyclin A and p57 expression during EVT differentiation revealed that approximately 36% of pCCT, 12% of dCCTs and 0.2% of EVTs are Cyclin A^+^ and p57^-^ (Fig 2F). To confirm de novo DNA synthesis in dCCTs and EVTs, we incubated floating explants of placental villi as well as of decidua basalis tissues with EdU for 18 hrs to study DNA incorporation *in situ* (Fig 2G and 2H). The degree of EdU incorporation was 55% in proximal HLA-G^-^ pCCTs, 20% in HLA-G^+^ dCCTs and 0.5% in HLA-G^+^ EVTs (Fig 2I). These data suggest that formation of HLA-G^+^ dCCTs is accompanied by the induction of endocycles, which markedly decline in decidual EVTs as noticed by strong induction of p57, downregulation of proliferation markers and lowered EdU incorporation.

### Decidual EVTs induce expression of SAβG and βG protein

**Fig 3:**
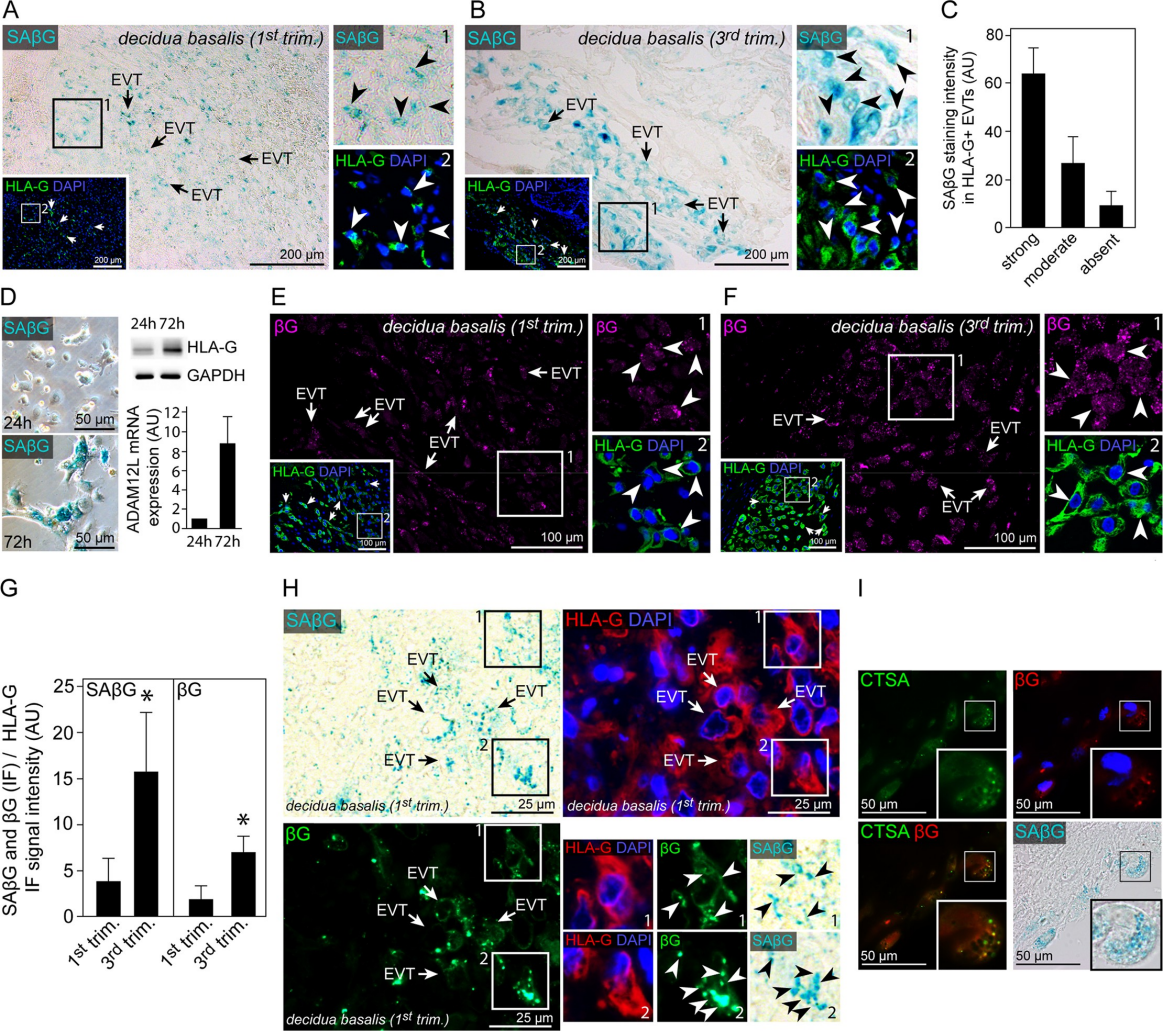
Senescence-associated β-galactosidase activity in HLA-G positive invasive, decidual EVTs co-localizes with beta-galactosidase protein and lysosomal cathepsin A. (A) Cryo-section of first trimester decidua basalis tissue (n = 7) showing co-stained SAβG activity (blue, large image) and HLA-G (green, insert). (B) Cryo-section of third trimester decidua basalis tissue (n = 6) showing co-stained SAβG activity (blue, large image) and HLA-G (green, insert). (C) Quantification of SAßG staining intensity in first trimester HLA-G^+^, deciudal EVTs. (D) SAβG activity in isolated primary human trophoblasts after 24 hrs (top) and 72 hrs (bottom) of cultivation (left panel). Western blot analysis (n = 3) of the same cultures showing increasing HLA-G^+^ and ADAM12L expression in differentiating EVTs after 72 hrs of cultivation. (E) Section of the same first trimester decidua basalis tissue (n = 3) depicted in A showing βG (magenta, large image) and HLA-G (green, boxed insert) co-staining. (F) Section of the same third trimester decidua basalis tissue (n = 3) depicted in B showing βG (magenta, large image) and HLA-G (green, boxed insert) co-staining. (G) Ratio of SaβG and βG relative to HLA-G signal intensities in first (n = 6) and third (n = 7) trimester decidua basalis tissue sections. (H) Section of first trimester paraffin-embedded decidua basalis tissue (n = 3) showing SAβG activity (blue), HLA-G (red) and βG (green) co-staining. (I) IF staining of SAβG (blue) expressing, decidual EVTs with antibodies against βG (red) and CTSA (green) (n = 3). The picture in the lower left corner represents a merged image of the green (CTSA) and red (βG) channel. Zoomed insets on the right show image details of the boxed area, arrowheads indicate SAβG (A and B) or βG (E and F) and HLA-G double positive cells. In (H) zoomed insets represent HLA-G, βG and SAβG triple positive regions marked by arrow heads. DAPI was used to visualize nuclei.

Although previous studies have shown signs for cellular senescence in syncytiotrophoblasts [35] no study has evaluated senescence-associated (SA) markers in EVTs. First, SA beta-galactosidase (βG) activity was determined in first and third trimester decidual basalis frozen tissue sections revealing intense SAβG signals in EVTs (Fig 3A). In contrast, CCTs showed very low SAβG activity (S3A Fig). Decidua stromal cells have recently been shown to exhibit SAβG activity and thus decidua parietalis tissue sections served as a positive control (S3B Fig) [36]. Strongest SAβG activity was found in third trimester EVTs (Fig 3B). Moreover, the majority of first trimester, decidual EVTs showed SAßG at a strong or moderate level, suggesting a global induction of a senescent phenotype in invasive trophoblasts (Fig 3C). Well in line, differentiated EVTs showed intense SAβG signals *in vitro* while early cultures of non-differentiated EGFR^+^ trophoblasts where mostly negative for SAβG (Fig 3D). In parallel, we stained paraffin-embedded tissue sections from the same patients with an antibody against βG (Fig 3E and 3F). Generally, βG protein expression intensity in first and third trimester tissue sections correlated well with SAβG activity in EVTs and CCTs (Fig 3 and S3C Fig). Quantification of both SAβG activity and βG protein expression revealed a significant induction in term EVTs when compared to first trimester sections (Fig 3G). Further IF stainings of cryo and paraffin embedded decidual tissue sections confirmed that SAβG activity and βG protein expression widely co-localize in EVTs (Figs 3H and S3E). Well in line, βG protein and the lysosomal marker cathepsin A (CTSA) are co-expressed in SAβG^+^, decidual EVTs (Fig 3I). Altogether, these data demonstrate induction of SAβG activity in invasive EVTs and confirm that SAβG activity likely reflects accumulation of lysosomal βG protein as previously suggested [37–39].

### EVTs induce a senescent phenotype and p57/cyclin E double knockdown reduces SAβG activity

**Fig 4:**
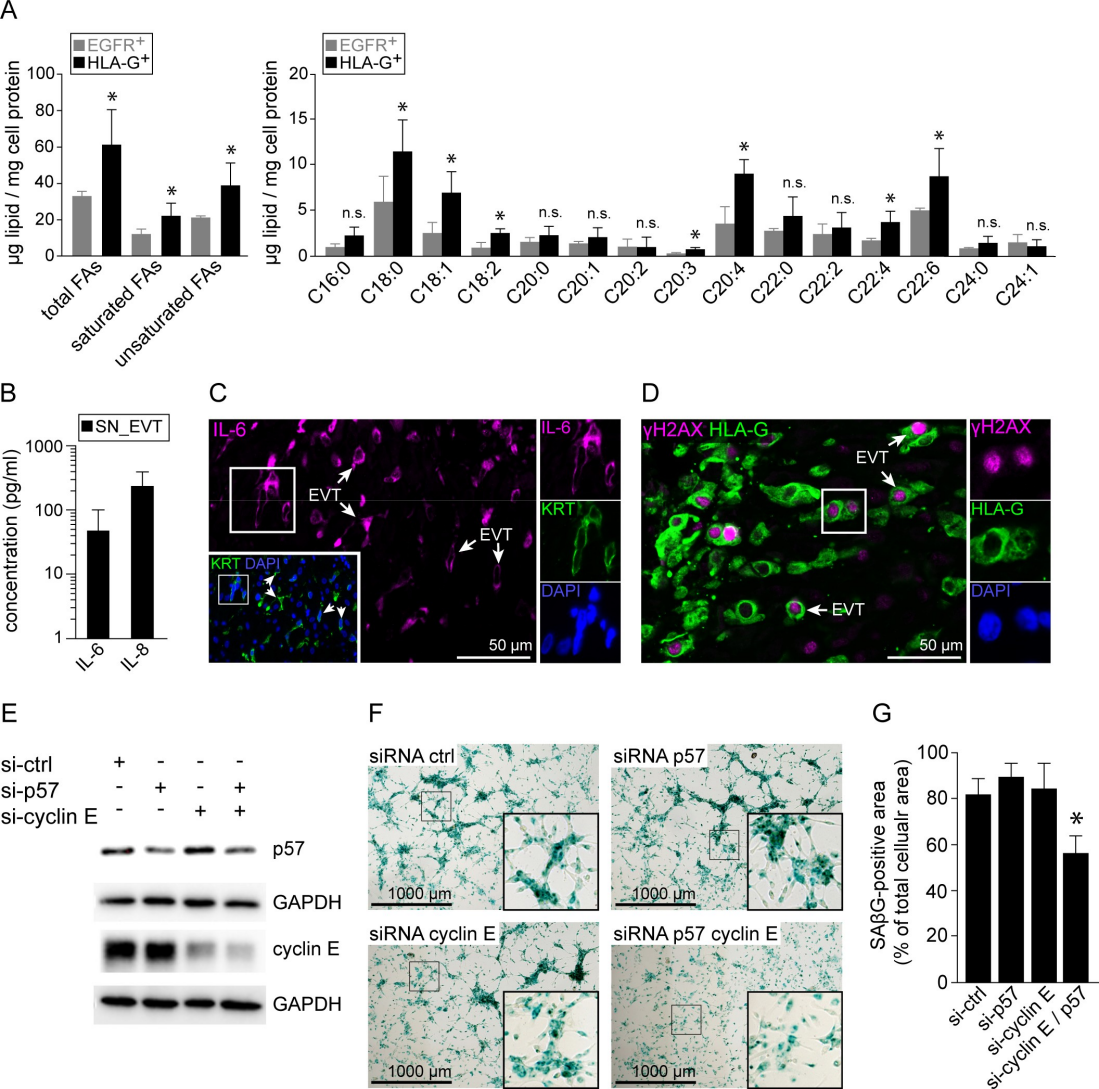
Decidual EVTs upregulate senescence-associated markers. (A) Gas chromatography-assisted analysis of fatty acid contents in isolated EGFR+ and HLA-G+ trophoblasts (n = 3). (B) Levels of IL-6 and IL-8 secreted by cultivated EVTs. (C) Section of first trimester decidua basalis tissue (n = 3) showing IL-6 (magenta, large image) and KRT co-staining (green, insert). (D) IF co-staining of paraffin embedded decidua basalis tissue sections (n = 3) with antibodies against γH2AX (magenta) and HLA-G (green). (E) Western blot analysis of p57 and cyclin-E single- and double-knockdowns (si-) performed in primary EVTs. Glyceraldehyde 3-phosphate dehydrogenase (GAPDH) served as loading control. One out of three independent experiments is shown. (F) SAßG expression in cultivated EVTs treated with siRNA targeting p57 or cyclin E. (G) Quantification of SAßG staining intensity in cultivated EVTs (n = 4). Zoomed insets on the right show image details of the boxed area, arrowheads indicate IL-6 (C) or γH2AX (D) and KRT or HLA-G double positive cells, respectively. DAPI was used to visualize nuclei.

Additionally, SA-associated metabolic and secretory phenotypes were analysed in isolated HLA-G^+^ and EGFR^+^ trophoblasts. First, electron microscopy-assisted analysis revealed pronounced glycogen storages within EVTs (S3D Fig). Using gas chromatography we determined cellular contents of triglycerides and fatty acid species in isolated vCTBs and EVTs. Firstly, EVTs display a trend towards increased triglyceride levels (S3F Fig). Analysis of total fatty acids revealed a significant increase in total fatty acids as well as different fatty acids species in these cells (Fig 4A). Additionally, transcripts of members of the SA secretory phenotype (SASP) were significantly upregulated in EVTs when compared to villous cytotrophoblasts (S3G Fig). Besides well-studied genes such as fibronectin, MMP2/3 or IGFBP3 we noticed induction of the two pro-inflammatory cytokines interleukin (IL) -8 and -6. Subsequent luminex-based measurement confirmed secretion of both IL-6 and IL-8 by cultivated EVTs (Fig 4B). In addition, EVT-associated expression of IL-6 was confirmed by IF stainings of first trimester decidua basalis tissues (Fig 4C). Similar analysis revealed induction of phosphorylated H2A histone family, member X (γH2AX) (Fig 4D), a well described marker for cellular senescence in non-malignant tissue [40]. To further study possible regulators of EVT-associated senescence we performed siRNA-mediated knockdown of *CDKN1C* and/or *CCNE1*, encoding p57 and cyclin E, respectively (Fig 4E). Strikingly, double knockdown of *CDKN1C* and *CCNE1*, significantly reduced expression of SAβG in cultivated, primary EVTs (Fig 4F and 4G). In summary, these data suggest that EVT invasion into the decidua is accompanied by the induction of cellular senescence.

### EVTs from complete hydatidiform moles show excessive expression of cell cycle markers, increased nuclear size and reduced signs for cellular senescence

**Fig 5:**
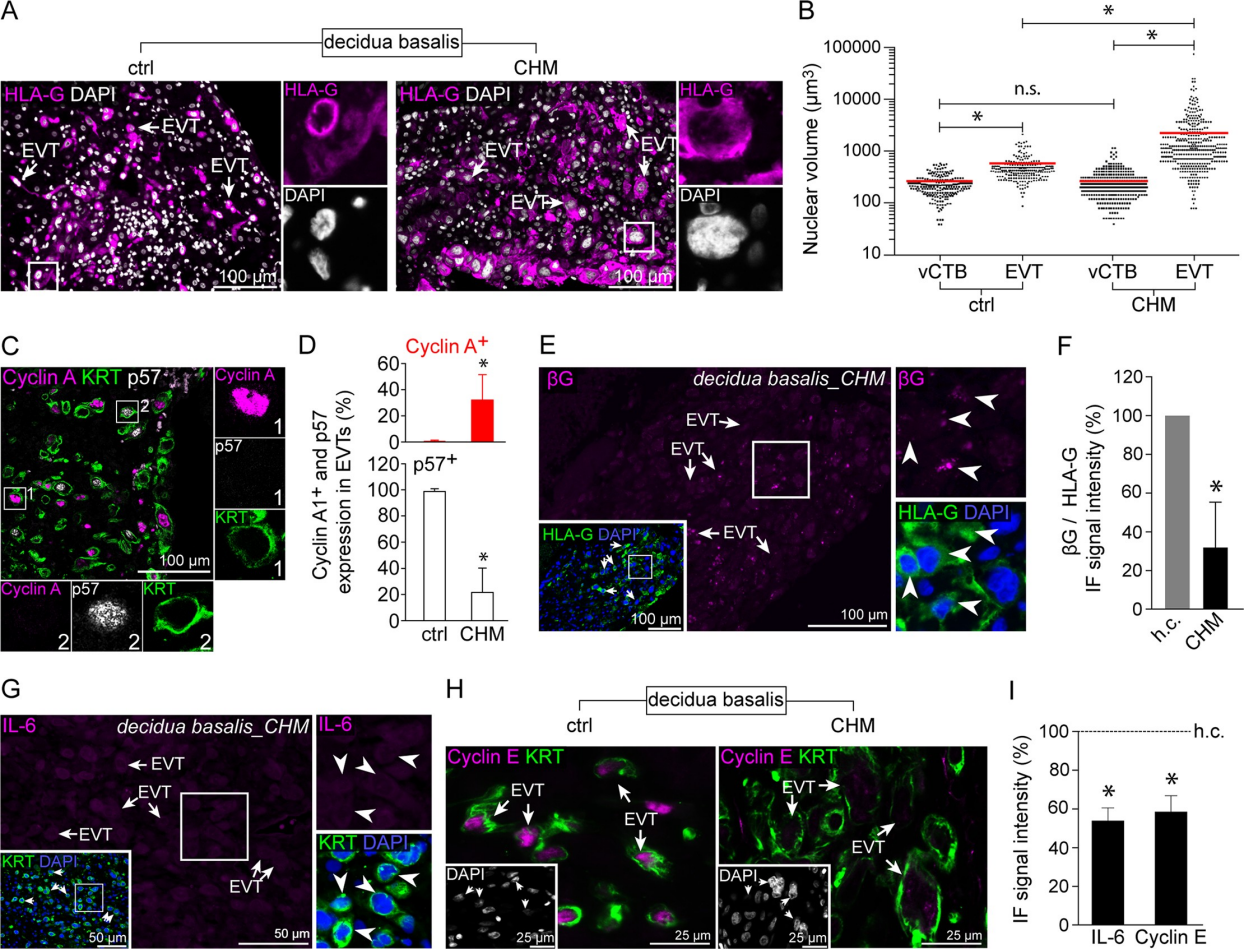
Complete hydatidiform mole EVTs exhibit increased ploidy and decreased cellular senescence. (A) IF co-staining of healthy (left image) and CHM (right image) first trimester decidua basalis tissues showing HLA-G^+^ (magenta) invasive EVTs. (B) Volumes calculated on the basis of the cross-section radius of vCTB and EVT nuclei of healthy (n = 20) and CHM (n = 23) diagnosed first trimester placentas. (C) IF triple-staining of first trimester decidua basalis tissue showing Cyclin A (magenta), p57 (grey) and Keratin7 (KRT7, green) in CHM-EVTs. (D) Rates of Cyclin A^+^ and p57^+^ EVTs in CHM (n = 18) and age-matched healthy control tissues (n = 15). (E) Section of CHM diagnosed first trimester decidua basalis tissue (n = 6) showing beta-galactosidase (βG) (magenta, large image) and HLA-G co-staining (green, insert). (F) Ratio of βG to HLA-G signal intensities in CHM diagnosed first trimester tissues (n = 23) age-matched healthy control sections (n = 15). (G) IF co-staining of CHM first trimester dedicuda basalis tissue showing IL-6 (magenta) and KRT (green, insert). (H) IF stainings of healthy (left image) and CHM (right image) decidua basalis tissues demonstrating cyclin E (magenta) and KRT (green) expression in invasive EVTs. (I) Ratio of IL-6 (n = 12) and cyclin E (n = 10) to KRT signal intensities in CHM diagnosed first trimester tissues age-matched healthy control sections (n = 22). Digitally zoomed insets display a split-channel-depiction of the boxed area. DAPI was used to visualize nuclei.

CHM is classified as a hypertrophic disease characterized by hyperproliferative villous cytotrophoblasts and cell column trophoblasts [41]. Therefore, we were interested to investigate whether EVTs are also affected by this hyperplastic condition. First we determined the nuclear size of CHM-EVTs and noticed a markedly increased nuclear volume in these cells (Fig 5A). When compared to age-matched healthy EVTs, the nuclear volume of CHM-EVTs was approximately 10-fold higher (Fig 5B) indicative for exacerbated polyploidization. Although, all cell types at the placental villus lack p57 expression we noticed induction of p57 in CHM-EVTs (S4A and S4B Fig). Triple-stainings revealed that the ratio between KRT7^+^/cyclin A^+^/p57^-^ and KRT7^+^/cyclin A^-^/p57^+^ was strongly shifted towards an endocycling phenotype in CHM placentas when compared to healthy controls (Fig 5C and 5D). Well in line, CHM-EVTs frequently express pRB (S4C Fig). Based on our previous finding we determined βG protein expression to analyse senescence in CHM-EVTs. These analyses revealed significantly reduced levels of βG when compared to age-matched healthy control sections (Fig 5E and 5F). Finally, EVT-associated IL-6 and cyclin E expression was diminished in cases of CHM (Fig 5G–5I). Altogether, these data reveal exacerbated polyploidization, markedly induced cell cycle marker expression and reduced signs for senescence in CHM-EVTs.

## Discussion

EVT differentiation is a multi-step process, which involves massive cell proliferation of EGFR^+^ pCCTs and formation of non-dividing HLA-G^+^ dCCTs that invade the maternal uterus upon contact with the decidua. Their equivalent trophoblast subtype in rodents, TGCs are well-characterized for their highly polyploid genome harboring regions with under- or–over-replicated domains [16, 17]. However, genomic alterations in human EVTs have been poorly elucidated. Some previous data described the whole genome content in human trophoblasts. Zybina et al. suggested polyploidization in human EVTs by measuring diameters of DAPI-stained nuclei in villous, decidual and myometrial trophoblasts [20]. They reported an up to 18N genomic status in myometrial EVTs. To more deeply characterize the genome of EVTs we performed various different methods including CLSM-guided 3D-construction of trophoblastic nuclei demonstrating that EVT differentiation is associated with a significant increase in nuclear volume. Subsequent determination of DNA content revealed a predominant tetraploid status in HLA-G^+^ CCTs and EVTs with no signs for CNVs. This finding contrasts cytogenetic analyses, including our own, reporting higher ploidy numbers, aneuploidies and gene amplification in human EVTs [18, 19]. However, FISH-based analyses of genomic and in particular centromeric regions in trophoblasts may be difficult to interpret as polyploid cells often show delayed or asymmetrical chromosomal separation, polyteny or diplochromosomal ploidy. Indeed, polyteny has been demonstrated in mouse trophoblasts [42, 43]. Asymmetrical chromosomal separation has been confirmed in polyploid human megakaryocytes [44] and is a common phenomenon in tetraploid cells [45]. Finally, genomic reduplication was suggested to result in diplochromosomes characterized by tightly associated quartets of centromeres and chromosome arms [46]. To circumvent this, we determined DNA content of isolated trophoblasts by FACS analysis and screened for CNVs by performing whole-genome sequencing (WGS) in combination with BIC-seq analysis. Here, the vast majority of HLA-G^+^ CCTs and EVTs showed a 4N status. Different to mouse TGCs, analysis of WGS data revealed no sings for CNVs in isolated HLA-G^+^ trophoblasts, suggesting that human EVTs fully replicate their entire genome. However, on the basis of our experiments we cannot exclude the presence of a small EVT subpopulation yet showing signs for CNVs and/or higher polyploidy rates. Therefore, further studies using single cell DNAseq approaches are required to address this uncertainty. Moreover, these studies led us to propose that HLA-G^+^ trophoblasts undergo active replication as these cells contain more DNA than EGFR^+^ trophoblasts. Indeed, a fluorescence-based reporter system indicated cycling trophoblasts at the distal end of outgrowing explant cultures. While, proximal EGFR^+^ CCTs showed proliferation marker expression including cyclin B, Ki67, pH3 and AuroraB we could not detect these cell division proteins in HLA-G^+^ CCTs. Of note, Ki67 expression has recently been shown to exert important functions during mitosis [47]. Instead, HLA-G^+^ CCTs expressed G/S-phase markers such as cyclin E and cyclin A along with strong induction of the cyclin-dependent kinase p57. Interestingly, cyclin E and p57 were shown to be essential for mouse TGCs polyploidization [13–15]. In mice, TGC endocycles display oscillating p57 and cyclin E/cyclin A levels of expression [11, 12]. Moreover, degradation of p57 is triggered by CCNA/Cdk2-dependent phosphorylation facilitating resetting of DNA replication in G1/S. Our data show that indeed cyclin A and p57 are reciprocally expressed in human HLA-G^+^ CCTs and EVTs suggesting that a CCNA^+^/p57^-^ expression profile is indicative for an active S-phase in these cells. Whether the human EVT-associated endocycle is indeed controlled by oscillating G/S phases as shown in TGCs or also involves other cell cycle phases needs to be addressed in future studies. Evaluation of CCNA and p57 co-expression as well as EdU incorporation during EVT differentiation revealed DNA replication in the distal cell column that sharply drops in EVTs. Given that we could not detect any signs for cell division in dCCTs we assume that S-phase activity in these cells finally results in a predominant tetraploid-phenotype in human EVTs. We detected only a very small number (approx. 1%) of EVTs that incorporated EdU in vitro or expressed cyclin A. In agreement with these data, we consistently detected a minor subpopulation containing a DNA content of > 4N in pCCTs and EVTs. Whether enhanced polyploidization in this small population of EVTs is linked with specific functionality and/or phenotype is currently under investigation. Nevertheless, the vast majority of EVTs seems to be in a growth arrested status. Interestingly, EVTs show high expression levels of cyclin E and p57, two markers indicative for cellular senescence in non-cycling cells [29, 30, 48, 49]. In addition, tetraploidy is believed to trigger cellular senescence in order to prevent excessive polyploidization, genomic instability and tumorigenesis [50, 51]. Senescence has traditionally been attributed with pathological alterations and loss of functionality such as aging. However, two recent scientific reports demonstrated a senescence-related function in mouse development [33, 34]. Senescent cells are non-proliferative, exhibit activation of SAβG, altered metabolic signatures and induce a SASP [24]. Surprisingly, we noticed strong induction of SAβG activity in EVTs as well as in differentiated EVTs in vitro whereas all other trophoblast subtypes showed no prominent signal, except some activity in the syncytium and in dCCTs. Moreover, we found that SAβG activity was more pronounced in EVTs at term. In this context, it might be that increased SAβG activity is a sign for loss of functionality. In addition, it is well-documented that cellular senescence induces self-targeted clearance by immune cells. For instance, oncogene-induced senescence in hepatocytes initiates their immune-mediated clearance to prevent malignancy [52]. This phenomenon is triggered by a Th1-polarized CD4+ T-cell response [53]. It is therefore tempting to speculate that while immune-mediated clearance of senescent EVTs is suppressed by the anti-inflammatory Th2-like decidual environment, senescence may protect from systemic spread of intact EVTs. In addition to SAβGal activity and protein levels we also characterized the specific senescence-associated phenotype of EVTs. Senescent cells often show a global change in their metabolism. This includes enhanced glycogen storage [54], induction of fatty acid synthesis [55] as well as secretion of inflammatory cytokines, chemokines, extracellular matrix (ECM)-associated factors and other signaling molecules including interleukin (IL)-6, IL-8, fibronectin, vascular endothelial growth factor and matrix metalloproteinases [32, 56, 57]. While an EVT-specific increase in glycogen and lipid content has been demonstrated [58], we show for the first time that EVTs formation is associated with an induction of fatty acid content. Since senescence cells increase in size [25] and in particular enhance their secretory activity, increased global fatty acid synthesis is likely reflected by a greater need for membrane synthesis. High levels and pronounced induction was observed for stearic acid (C18:0), oleic acid (C18:1) arachidonic acid (C20:4) and docosahexaenoic acid (C22:6). Oleic, arachidonic and docosahaexaenoic acid may also impact on EVT function as they were shown to induce tube formation in HTR-8 cells and/or survival [59, 60]. Arachidonic acid is the precursor of eicosanoids including prostaglandins and leukotrienes. Interestingly, prostaglandins induce uterine vasodilation [61] or promote Th2 and regulatory T-cell responses [62]. Leukotriene B4 is a well described leukocyte chemoattractant including recruitment of T-cells [63] and neutrophils [64]. The latter have recently been suggested to show a pro-angiogenic phenotype in the human deciuda [65]. We further found that EVTs secret IL-6 and -8, two prominent members of the SASP [32]. Since both cytokines are well-described for their crucial role in neutrophil recruitment [66–68], EVT-mediated release of IL-6 and -8 might support vascular remodelling during pregnancy. Along these lines, IL-6 has been shown to potently suppress vascular smooth muscle contraction [69]. We also detected a positive staining for γH2AX indicating DNA double strand breaks in EVTs, a well described trigger of cellular senescence [40]. In this context, it is interesting to note that overexpression of cyclin-E causes DNA damage [70, 71] and was shown to induce cellular senescence [48, 72]. Although, knockdown of cyclin E alone was not sufficient to alter induction of senescence in EVTs combined suppression of cyclin E and p57 significantly reduced SAβG activity in cultured EVTs. Since p57 has also been shown to induce cellular senescence in human cancer cells [29, 30] and vascular smooth muscle cells in mice [73] we propose a role p57 and cyclin E in the induction of the EVT-associated senescent phenotype in humans. The fact that both cell cycle regulators are also expressed in non-senescent trophoblasts points towards multifaceted roles for p57 and cyclin E in placental development. Available data suggest that increased expression of cyclin E and p57 beyond physiological levels are necessary to induce cellular senescence. Similar regulatory mechanisms are likely to occur during EVT differentiation since p57 [21] is upregulated during EVT differentiation and published microarray and RNAseq data also suggest induction of cyclin E transcripts [74, 75].

CHM placentas are characterized by a hyperplastic, androgenetic placental phenotype. Hyperproliferation noticed in vCTBs and pCCTs [41] is likely caused by a complete lack of growth restricting maternally expressed genes [76]. Indeed, cases of Beckwith-Wiedemann syndrome characterized by loss of p57 expression or mutations in *CDNK1C* [77] share several pathological features in placental development with CHM such as hyperplasia or excessive EVT formation [78, 79]. Whether the hyperplastic phenotype in CHM also affects cell cycle of EVTs has not been studied so far. Interestingly, we noticed a highly enlarged nuclear volume in EVTs of CHM placentas suggesting exacerbated endocycles in these cells. In addition, we found that EVTs of CHM placentas induce p57 confirming previous reports demonstrating that haploid, androgenic mole placentas express paternally imprinted genes such as *H19* [80] and CDKN1C [81], normally expressed from the maternal allele. Despite reactivation of p57 we also found a pronounced endocycle-associated phenotype characterized by markedly elevated numbers of p57^-^/cyclin A^+^ EVTs. Unfortunately, we were not able to determine SAβG activity in CHM since endogenous enzyme activity can only be measured in fresh tissues. Nevertheless, our data show that SAβG activity strongly correlates with EVT-associated overexpression of lysosomal βG protein when compared to other trophoblast subtypes. We therefore suggest that reduced βG protein expression in CHM-EVTs is indicative for a suppressed senescent phenotype. This conclusion is further supported by significant lower levels of IL-6, p57 and cyclin E in these cells.

This suggests that the hyperplastic phenotype noticed in CHM extends to EVTs, which show excessive endocyclic activity characterized by hyperpolyploidization and suppressed senescence-induced growth arrest. As discussed above, multiple endocycles in mouse TGCs result in CNVs. It is tempting to speculate that repeated DNA replication in the absence of mitosis might also result in a gradual disorganization of the DNA methylation at CpG islands in EVTs, as it has been demonstrated in rodent TGCs. Along these lines, hyperpolyploidization in CHM-EVTs could lead to loss of epigenetic methylation marks and therefore reactivate expression of p57 and H19. Indeed, partial escape from X chromosomal inactivation together with reduced promotor methylation of X-linked genes has been noticed in endreduplicating mouse TGCs [82].

**Fig 6:**
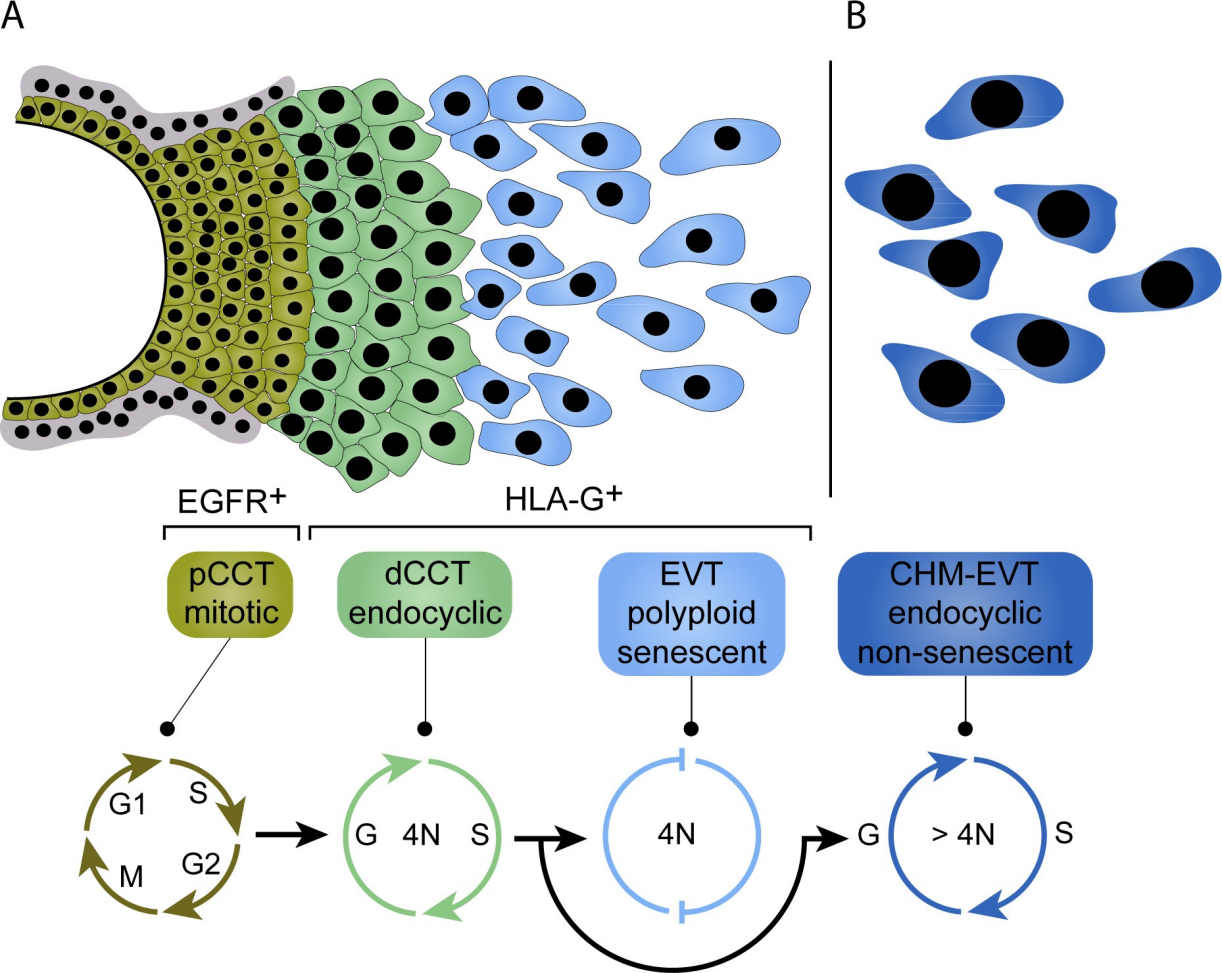
Proposed model of tetraploidization in HLA-G^+^ trophoblasts and induction of senescence during EVT differentiation. (A) During early pregnancy, EGFR^+^ pCCTs form a highly proliferative so-called cell column, which give rise to HLA-G^+^ CCTs at the distal end of the cell column. During this transition, a endoreduplicative cell cycle provokes tetraploidization in dCCTs. Endoreduplication in human HLA-G^+^ trophoblasts is likely characterized by an cyclin A^+^/p57^-^ expression pattern, expressed by a subset of dCCTs. Upon invasion into the deciuda, EVTs lose their cyclic activity marked by a dominant cylclin A^-^/p57^+^ phenoptye and undergo cellular senescence. Induction of cellular senescence is at least partly regulated by p57 and cyclin E. (B) The balance between endocycle and senescence is disturbed in CHM. CHM-EVTs show exacerbated endocyclic activity characterized by an increase in cyclin A^+^/p57^-^ EVTs, enhanced polyploidization and reduced signs for cellular senescence.

In summary, this report is the first to show that induction of EVT differentiation is accompanied with induction of endocyclic activitiy and tetraploidization (Fig 6A). We suggest a Cyclin A+/p57- expression profile as indicative marker for endoreduplicating HLA-G+ trophoblasts. However, upon invasion into the decidua EVTs exit the replicative cell cycle followed by growth arrest and induction of cellular senescence. In contrast, hyperplastic CHM-EVTs continue their endoreduplicative cell cycle likely leading to repression of senescence and as a consequence exacerbated polyploidization (Fig 6B).

## Material and methods

### Tissue collection

Placental and decidual tissues (6–12^th^ week of gestation, n = 85) were obtained from legal, elective pregnancy terminations. CHM placentae (n = 23) were obtained from the archive of the Clinical Institute of Pathology, Medical University of Vienna, Austria. Diagnosis of CHM was based on the original report including high resolution ultrasound, determination of the proliferative index and human gonadotropin beta levels as well as absence of p57 expression in placental villous cytotrophoblasts. Utilization of tissues and all experimental procedures were approved by the local Ethics Committee of the Medical University of Vienna, Austria. Methods were carried out in accordance with the approved guidelines. Written informed consent was obtained from all patients.

### Isolation and cultivation of primary trophoblasts

Cytotrophoblasts (CTBs) were isolated by enzymatic dispersion and Percoll density gradient centrifugation (10–70% (vol/vol); GE Healthcare) of pooled first trimester placentas (*n* = 2–5 per isolation) as described in [83] and plated (45 min) in culture medium (DMEM/Ham’s F-12, 10% (vol/vol) FCS, 0.05 mg/mL gentamicin, 0.5 μg/ml fungizone; Gibco) allowing for adherence of contaminating stromal cells. Nonadherent trophoblasts were collected and seeded in culture medium onto fibronectin-coated (20 μg/mL; Millipore) dishes (2.5 × 10^5^ cells per square centimetre). The contamination with stromal cells was routinely tested by IF with antibodies detecting cytokeratin 7 (trophoblast cells) and vimentin (fibroblasts). Vimentin-positive cells were < 3%. CTBs were cultured for up to 72 h with media changed after 20h. EGFR^+^ trophoblasts were separated from HLA-G^+^ trophoblasts by magnetic-activated cell sorting, using EGFR-PE and HLA-G-PE antibodies, which were labelled with anti-PE micro-beads (Miltenyi Biotec).

### Separation of primary extravillous trophoblasts

Decidua basalis tissue was minced into ~3 mm^3^ pieces and digested under agitation in 2 mg/ml collagenase I (Life Technologies) and 0.5 mg/ml DNase I (Sigma Aldrich) in HBSS containing 25 mM HEPES (Life Technologies) for 30 min at 37°C. Dispersed cells were pooled and filtered through a 70 μm cell strainer. To label extravillous trophoblasts, cells were incubated with HLA-G-PE antibodies for 20 min at 4°C and prepared for flow cytometric analysis as described below.

### Immunofluorescence of placental and decidual tissues

First trimester placental and decidual tissues (6^th^ - 12^th^ week of gestation) were fixed in 7.5% (wt/vol) formaldehyde and embedded in paraffin. Serial sections (3 or 30 μm) were deparaffinised in Xylol (10 min) and rehydrated in a decreasing series of ethanol (100%, 90%, 70%, 0%; 1 min each step). Antigen retrieval was performed using 1× PT module buffer 1 (pH 6, Thermo Fisher Scientific) for 35 min at 93°C using a KOS microwave histostation (Milestone). Sections were blocked using 0.05% cold water fish skin gelatine (Sigma-Aldrich) for 30 min at room temperature and incubated overnight at 4°C with primary antibodies in PBS with 0.05% fish skin gelatine (see antibody list). Secondary antibodies were incubated for 45 min at room temperature in PBS with 0.05% fish skin gelatine and 1 μg/ml 4′,6-Diamidin-2-phenylindol (DAPI, Roche). Finally, tissue sections were mounted with Fluoromount-G (Thermo Fisher Scientific) and covered. Images were acquired with a fluorescence microscope (Olympus BX50 equipped with Cell^P software) or with a confocal laser scanning microscope (LEICA, TCS SP8X, equipped with Leica LAS AF software). Confocal images are depicted as maximum projection of total z-stacks and brightness and contrast were adjusted in a homogenous manner using Leica LAS AF.

### Real-time PCR

RNA isolation, reverse transcription and qPCR analyzes were performed as described previously [83] using TaqMan Gene Expression Assays: ADAM12L (Hs 00185774_m1). Signals (ΔCt) were normalized to TATA-box binding protein (TBP) (ABI, 4333769F).

### Western blotting

Protein extracts were immobilized on PVDF membranes and incubation with primary and secondary antibodies (S1 Table) was performed as published [41]. Signals were developed using ECL prime detection Kit (GE Healthcare) and visualized with FluorChemQ imaging system (Alpha Innotech). Signal quantification was performed using Image J software.

### siRNA transfection

Freshly isolated trophoblasts (see above) were transfected with SMARTpoolON-TARGETplus siRNAs (GE Dharmacon) using Lipofectamine RNAiMAX reagent (Invitrogen, Life Technologies) and cultivated for 48 hrs. The following siRNAs against the indicated mRNAs were used at a concentration of 40 nM: CDKN1C (L-003244-00-0005), CCNE1 (L-003213-00-0005) and non-targeting control (D-001810-10-05). After 48 h, transfected cells were subjected to western blotting. Alternatively, cells were fixed in 1x fixative solution (Cell Signaling) for 10 min at room temperature, washed 3 times with PBS and incubated overnight at 37°C with the β-galactosidase staining solution at pH 6.0 (Cell Signaling).

### Evaluation of p57/CCNA1 expression and EdU incorporation

In total, 1620 images were analyzed quantitatively. P57 and CCNA1 positivity in CCTs was analyzed by evaluating 15 tissue sections per sample (n = 10). Consecutive tissue sections were stained with antibodies against EGFR+ and HLA-G+ to determine proximal and distal regions of the CC. P57 and CCNA1 expression in KRT7+ EVTs was determined by evaluating 12 tissue sections per sample (n = 9). EdU incorporation into HLA-G+ dCCTs and EVTs, respectively was analyzed by evaluating 8 tissue sections per sample (n = 4). To ensure objectivity, the quantification of all IF stainings was analyzed by two independent investigators.

### 3D reconstruction of trophoblast nuclei

Image stacks were obtained using confocal laser scanning microscopy with 0.2 μm steps in z-axis of 30 μm tissue sections. 3D reconstruction was performed from DAPI signals using Imaris software (Bitplane AG). Nuclei that had been cut and were incomplete were disregarded from analysis. Volumes of nuclei were calculated and compared. 3D reconstructions were performed over five cell columns of three different placentas. Proximal cell column trophoblasts (CCTs) were defined as CCTs within 30 μm from the vCTB layer, distal CCTs were defined as CCTs within 60 μm from the most distally recorded CCTs.

### FUCCI cell cycle sensor analyses in placental explant cultures

First trimester placental villous explants (7^th^ - 9^th^ week of gestation, n *= 12*) were dissected under the microscope as mentioned in [83] and seeded onto collagen I drops (BD Biosciences, mixed with 10× DMEM and 7.5% sodium bicarbonate). One explant per 48 well was seeded and incubated for 6 h at 37°C to allow anchorage. Explants were then mounted with 200 μl DMEM/Ham's F-12 medium and 2.5 μg/ml fungizone (Invitrogen) and incubated over night at 37°C. The next day, media was discarded and 160 μl media supplemented with 20 μl Premo geminin-GFP reagent and 20 μl Premo Cdt1-RFP reagent (2×10^6 particles of each per explant) were added according to manufacturer’s protocol (Thermo Fisher Scientific) to those explants showing trophoblast outgrowth and incubated for an additional 24 h. Finally, media was discarded and explants were either mounted with prewarmed PBS and immediately digitally photographed using the EVOS FL Color Imaging System or fixed (4% PFA, 15 min, 4°C), permeabilized (0.1% Triton X-100, 5 min, 4°C) and stained (DAPI, 10 min, room temperature) in the dark and then digitally photographed.

### FISH

Isolated trophoblast were centrifuged and incubated with 0.5% KCL and incubated at 37°C for 20 minutes. Subsequently, cells were treated with ice-cold fixative (one part acetic acid and three parts methanol) and incubated for 10 minutes at -20°C. Fixed cells were air-dried on glass slides at 42°C for 15 minutes and treated with pepsin (350 μl 0.5% Pepsin with 1 ml 1N HCl ad 100 ml H2O) for 15 min at 37°C. Slides were then washed in PBS and PBS/20mM MgCl2 each 5 minutes at room temperature. Subsequent to dehydration in a 10% formaldehyde solution, cells were incubated with a “ready to use” solution containing centromer specific probes for X- and Y-chromosomes (DXZ1 (green) and DYZ3 (red) (Leica Biosystems). Hybridization was performed using a ThermoBrite system (Leica) for 16 h at 37°C after 5 minutes denaturation at 75°C. Afterwards, cells were washed for two minutes with 0.4x SSC with 0.3% NP40 followed by a 1 minute wash step with 2x SSC with 0.1% NP40. Slides were air-dried and covered with Vectashield Mounting Medium containing DAPI (Vector Laboratories Burlingame, USA). FISH signals were detected using an Axioplane2 imaging system (Zeiss) equipped with “MetaSystems Isis” software version 5.3.18.

### Flow cytometry

CTBs were isolated as described above and labelled with the FITC- and PE-conjugated antibodies outlined in S1 Table for 20 min at 4°C. Appropriate isotype-specific control antibodies were used accordingly. Then, cells were fixed (4% PFA, 15 min, 4°C), permeabilized (0.1% Triton X-100, 3 min, 4°C) and stained (DAPI, 10 min, room temperature) in the dark. Data were acquired on a FACScan flow cytometer (BD Biosciences) and analysed using FlowJo 7.6.5 software (Tree Star, Ashland, OR). Doublet discrimination was performed by plotting the area (FL-A) of the fluorescence light pulse against the width (FL-W).

### Gas chromatography

Lipids were isolated from cell pellets by standard Folch extraction. An aliquot of the pellet was used for cell protein determination by the Bradford assay. Triglycerides were directly analyzed by GC as described [84]. In brief, lipids were separated using a GC-2010 gas chromatograph (Shimadzu) equipped with a programmed temperature vaporizer injector and a ZB-5HT capillary column (15 m x 0.32 mm x 0.1 μm; Phenomenex). Trinonadecanoin (Sigma) was used as standard. For fatty acid analysis, FOLCH-extracts were trans-esterified using boron trifluoride-methanol solution (Sigma) at 80°C for 2 hrs followed by extraction with hexane. Lipids were separated on a ZB-FFAP capillary column (15 m x 0.32mm x 0.25 μm; Phenomenex) using pentadecanoin (Sigma) as standard. Chromatograms were analyzed using GC Solutions 2.3 (Shimadzu) and values were normalized to cell protein.

### Analysis of cytokines with multiplex bead assay

Multiplex bead assay kits were used according to the manufacturer’s protocol (Millipore) to determine the levels of IL-6 (detection limit 3.1 pg/ml) and IL-8 (detection limit 1.6 pg/ml) in culture supernatants from EVT cells. The analyses were performed using the Luminex200 IS system (Millipore) and the MasterPlex QT 2010 software (MiraiBio). Values below the detection limit were given half the value of the detection limit and the concentration in the corresponding control CM was subtracted from the concentration measured in the cell supernatants.

### DNAseq and BICseq

DNA isolated from EGRF^+^ and HLA-G^+^ human placental trophoblasts from two different donors (11^th^ and 12^th^ week of gestation) were sent to the Macrogen Laboratory for WGS. Two libraries were made for each sample/cell type using the TruSeq Nano Kit (Illumina). 150 bp paired-end read sequencing was performed on the HiSeq X Ten (Illumina), resulting in approximately 30X coverage for each library (~60X coverage for each sample). Sequence data was mapped to human reference genome hg19 using Burrows-Wheeler aligner [85]. Mouse placental WGS data was retrieved from BioProject accession number PRJNA213010 [17]. To determine whether there is copy number variation (CNV) in polyploid placental cells, data (sorted bam files) were analyzed using BIC-seq [86] for paired data with lambda = 4, following [16]. We also applied the HugeSeq pipeline that integrates four algorithms, including Pindel, CNVnator, BreakDancer and BreakSeq to discover CNVs, and we did not detect any high confidence CNVs that are unique to the polyploid placental cells [87–91].

### Evaluation of nuclear volume

Tissue sections (3 μm) of paraffin-embedded placental and decidual tissues were obtained and stained as described above. Mean diameters of DAPI signals were determined of epithelial vCTBs and invasive EVTs. For determination of the volume a perfect sphere (V = 4/3 × π × r^3^) was assumed for all nuclei.

### Analysis of cyclin E, IL6, SAβG and βG staining intensities

Images were taken using the Leica confocal microscope TSC SP8. ImageJ software was used to measure stained area (SAβG, βG) in relation to the HLA-G or KRT signal by analysing staining intensities at a predefined threshold. Cyclin E and IL-6 expression in EVTs was analysed using ImageJ software, by creating a mask containing all KRT+ cells and measuring the mean intensity of the respective signal within the mask. KRT staining intensity within the mask was used as a calibrator. To quantify the percentage of SAβG^+^ EVTs fluorescence and bright filed images were overlaid in Photoshop (CS6 Extended) and HLA-G^+^ areas were selected using the Magic Wand tool. The bright filed channel was then isolated and pre-selected HLA-G^+^ areas were cropped. Intensities were classified into strong, moderate and absent as illustrated in S5 Fig. SAβG expression in isolated EVTs was determined relative to total cellular area using ImageJ software.

### In situ β-galactosidase activity assay

SAβG activity was determined in first and third trimester placental and decidual (6^th^ - 12^th^ week of gestation) cryosections using the senescence β-galactosidase staining kit (Cell Signaling) and by adapting published protocols [33, 34]. Briefly, tissues were preserved in OCT compound, sectioned (4 μm) and fixed in 1x fixative solution (Cell Signaling) for 10 min at room temperature, washed 3 times with PBS and incubated overnight at 37°C with the β-galactosidase staining solution at pH 6.0. Subsequently, slides were counterstained with antibodies against HLA-G, ßG and DAPI. Alternatively, SAßG staining was performed in whole-mount first trimester placentas using the Senescence ß-Galactosidase Staining Kit. Briefly, whole-mount placentas were fixed at 4°C over night with 1X Fixative Solution, washed 3 times in PBS and incubated over night at 37°C with ß-Galactosidase Staining Solution (pH6). Placentas were subsequently dehydrated and perfused with Paraplast X-TRA (Sigma, St.Louis,MO; USA) using a KOS Microwave Histostation (Milestone, Sorisole; Italy), then embedded in paraffin for serial sectioning and counterstained with antibodies against HLA-G, ßG and DAPI.

### Accession codes and data availability

BioProject accession number for WGS of human trophoblastic samples: PRJNA445189

### Statistics

Statistical analysis was performed with Student’s unpaired t-test using SPSS 18 (SPSS Inc.). Gaussian distribution and equality of variances were examined with Kolmogorov–Smirnov test and Levene test, respectively. Comparisons of multiple groups were evaluated with one-way ANOVA and appropriate post hoc tests. A P-value of < 0.05 was considered statistically significant.

## Supporting information

S1 Fig HLA-G expression correlates with increased ploidy of human trophoblasts. (A) IF co-staining of first trimester placental tissue showing EGFR (green) and HLA-G (magenta) in cell column trophoblasts (n = 3). (B) DAPI staining of sorted primary isolated human trophoblasts using anti-EGFR and anti-HLA-G antibodes and magnetic-activated cell sorting (MACS) to compare nuclear size. Keratin7 (green) staining was used to confirm trophoblasts. (n = 3) (C) Quantification of the nuclear diameters of isolated trophoblasts from B. (D) Representative picture of the FISH analysis with probes against sex chromosomes X and Y (n = 3) (E) FISH analysis using probes against sex chromosomes X and Y of MACS-sorted HLA-G+ EVTs from decidua basalis tissue. (F) BIC-seq indicating CNVs by comparing a test (upper two panel: HLA-G+ EVTs, lower panel: mouse TGCs) and control genome using the statistical program BIC-seq. For BIC-seq, presence of the Y-chromosome is represented as elevated copy number compared to the other chromosomes Digitally zoomed insets display a split-channel-depiction of the boxed areas. DAPI (A and B, grey; D, blue) was used to visualize nuclei.
(TIF)

S2 Fig Human trophoblasts switch from a mitotic- to an endo-cycle as they differentiate. (A) FUCCI cell cycle sensor analyses of outgrowing placental explants. Lower left corner (placental villi (PV) are indicated by a red, dotted line). A representative outgrowth-area is shown in detail (indicated by a rectangle in the lower left picture). (B) IF co-staining of first trimester placental tissue showing phospho-histone 3 (pH3, magenta) and Aurora B (green) positive mitotic figures in vCTBs and proximal cell column (pCCT) trophoblasts. (C) IF co-staining of a first trimester placental consecutive tissue section presented in Fig 2C showing pH3 (magenta) and EGFR (green) expression of vCTBs and pCCTs. (D—F) IF co-staining of a first trimester placental tissue section showing p27 (D), p21 (E) and p16 (F) (magenta) and KRT7 (green) expression in EVTs. (G) IF co-staining of a first trimester placental tissue section showing HLA-G (magenta) and Cyclin A (green) expression of pCCTs and dCCTs. (H) IF co-staining of first trimester placental tissue showing Cyclin E (magenta) and p57 (green) expression of vCTBs and CCTs. (I) IF co-staining of first trimester decidual tissue showing Cyclin E (magenta) and p57 (green) expression of EVTs. Inset shows HLA-G (green) and DAPI (blue) staining of the same area. Digitally zoomed insets display a split-channel-depiction of the boxed areas. (blue) was used to visualize nuclei.
(TIF)

S3 Fig EVTs express markers of senescence. (**A**) Cryo-section of first trimester placental tissue showing co-stained SAβG activity (blue, large image) and Keratin7 (KRT7) (magenta, insert). Zoomed insets on the right show image details of the boxed areas, arrowheads indicate SAβG and KRT7 (red) positive trophoblast cells. (**B**) Cryo-section of first trimester decidua basalis tissue showing co-stained SAβG activity (blue, large image) and Keratin7 (KRT7) (magenta, insert). Zoomed insets on the right show image details of the boxed areas, arrowheads indicate SAβG and/or KRT7 (red) in decidual gland cells (left panel) and decidual stromal cells (right panel). (**C**) IF co-staining of first trimester placental tissue showing beta-galactosidase (βG, magenta, large image) and HLA-G (green, insert) co-staining. Zoomed insets on bottom show image details of the boxed area. (n = 3) (**D**) Representative electron microscopy image showing MACS-sorted, HLA-G^+^ primary human trophoblasts. (**E**) Section of first trimester cryo-embedded decidua basalis tissue (n = 3) showing SAβG activity (blue), HLA-G (magenta) and βG (green) co-staining. (**F**) Gas chromatography assistested analysis of triglyceride contents in isolated EGFR^+^ and HLA-G^+^ trophoblasts (n = 3). (**G**) Scatter blot (left panel) and table indicating significantly regulated SASP-associated genes in isolated EGFR+ and HLA-G+ trophoblasts. DAPI (blue) was used to visualize nuclei.
(TIF)

S4 Fig CHM-EVTs re-express p57. (A-B) IF co-staining of first trimester CHM placental tissues showing p57 (magenta) and KRT7 expression (green) in villous trophoblasts (A) and decidual EVTs (B). (C) Representative IF co-staining showing pRB (magenta) and KRT7 (green) expression in CHM-EVTs. DAPI (blue) was used to visualize nuclei. Digitally zoomed insets display a split-channel-depiction of the boxed area.
(TIF)

S5 Fig Quantification of SAβG expression in deciudal HLA-G^+^ EVTs. (A) Cryo-sectioned decidua basalis tissues were treated with SAβG activity assay and counterstained with an antibody against HLA-G (a-b). (B) Subsequently, fluorescence and bright field images were overlaid in Photoshop (c) and HLA-G^+^ areas were selected using the Magic Wand tool (d). (C) The bright field channel was then isolated (e) and pre-selected HLA-G^+^ areas were cropped (f). (C) Finally, SAβG signals were quantified as indicated in (g).
(TIF)

S1 Table List of all primary and secondary antibodies used for immunofluorescence of paraffin sections (IF-P), Western blotting (WB), magnetic active cell sorting (MACS) and flow cytometry (FC). (PDF)
