# Supplementary figures and images for: Genome amplification and cellular senescence are hallmarks of human placenta development
Source: PLoS Genet. 2018 Oct 12;14(10):e1007698. doi: 10.1371/journal.pgen.1007698 (PMC6200260; doi:10.1371/journal.pgen.1007698)

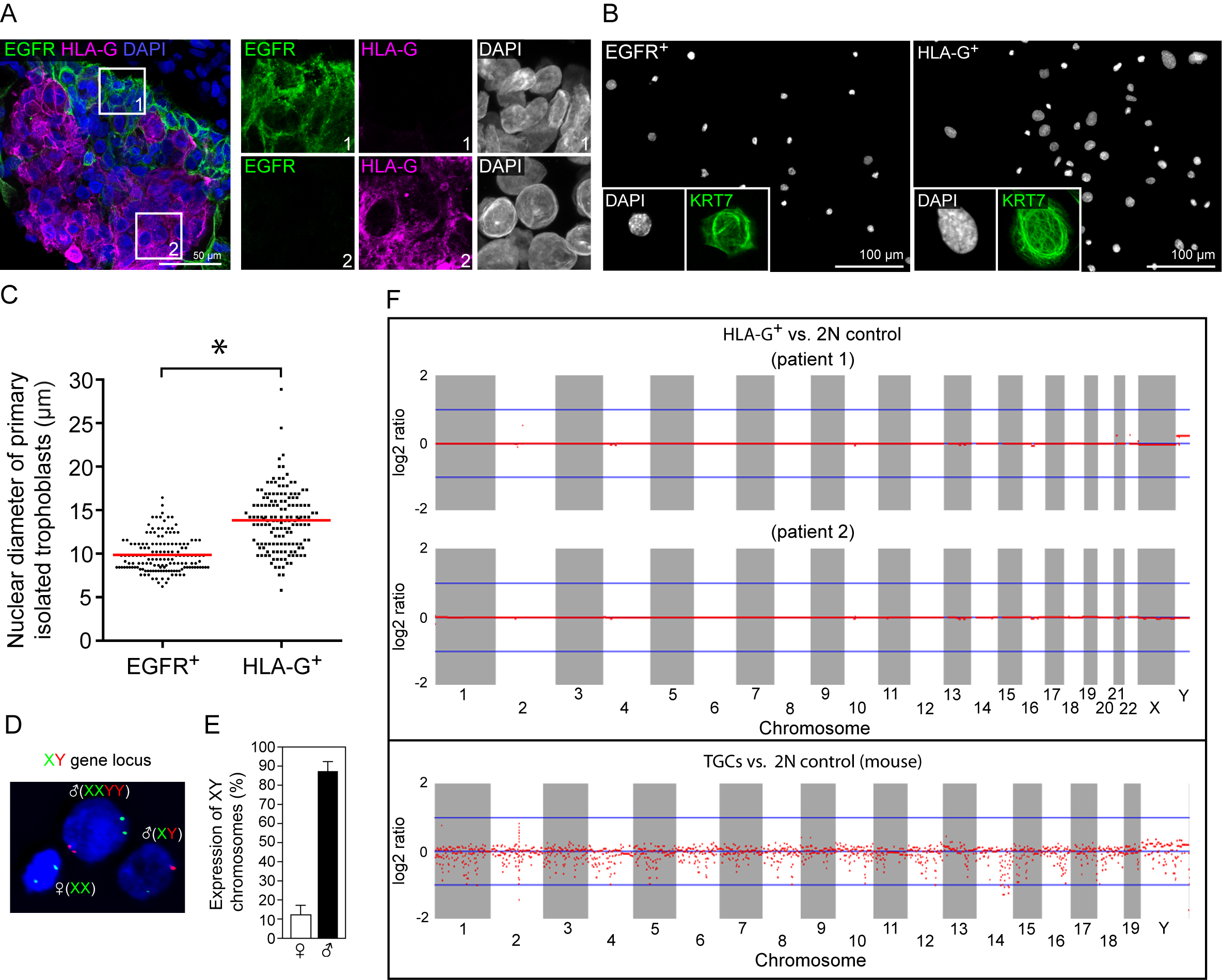

Supplement: S1 Fig — (A) IF co-staining of first trimester placental tissue showing EGFR (green) and HLA-G (magenta) in cell column trophoblasts (n = 3). (B) DAPI staining of sorted primary isolated human trophoblasts using anti-EGFR and anti-HLA-G antibodes and magnetic-activated cell sorting (MACS) to compare nuclear size. Keratin7 (green) staining was used to confirm trophoblasts. (n = 3) (C) Quantification of the nuclear diameters of isolated trophoblasts from B. (D) Representative picture of the FISH analysis with probes against sex chromosomes X and Y (n = 3) (E) FISH analysis using probes against sex chromosomes X and Y of MACS-sorted HLA-G+ EVTs from decidua basalis tissue. (F) BIC-seq indicating CNVs by comparing a test (upper two panel: HLA-G+ EVTs, lower panel: mouse TGCs) and control genome using the statistical program BIC-seq. For BIC-seq, presence of the Y-chromosome is represented as elevated copy number compared to the other chromosomes Digitally zoomed insets display a split-channel-depiction of the boxed areas. DAPI (A and B, grey; D, blue) was used to visualize nuclei. (TIF) [file pgen.1007698.s001.tif]

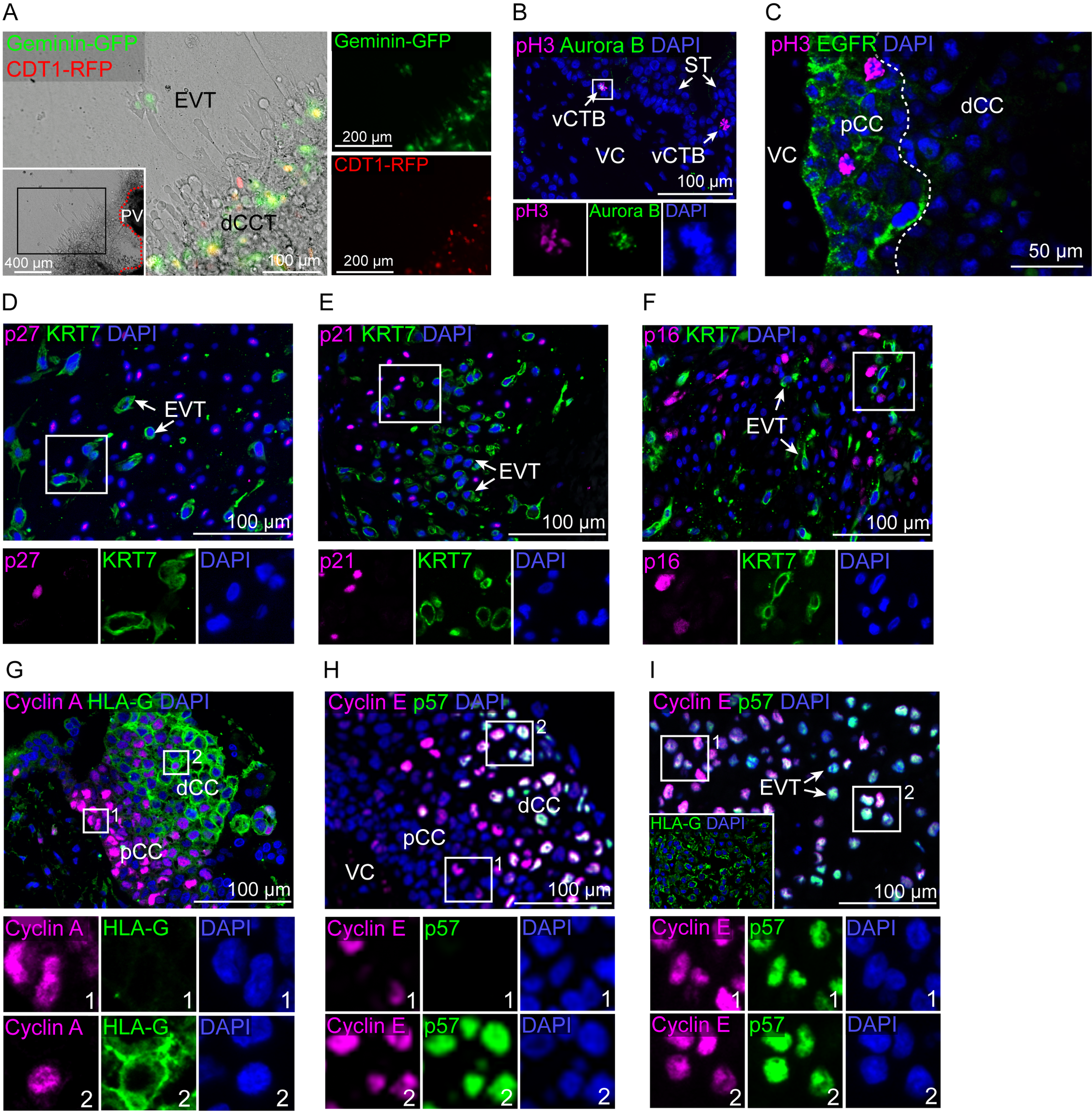

Supplement: S2 Fig — (A) FUCCI cell cycle sensor analyses of outgrowing placental explants. Lower left corner (placental villi (PV) are indicated by a red, dotted line). A representative outgrowth-area is shown in detail (indicated by a rectangle in the lower left picture). (B) IF co-staining of first trimester placental tissue showing phospho-histone 3 (pH3, magenta) and Aurora B (green) positive mitotic figures in vCTBs and proximal cell column (pCCT) trophoblasts. (C) IF co-staining of a first trimester placental consecutive tissue section presented in Fig 2C showing pH3 (magenta) and EGFR (green) expression of vCTBs and pCCTs. (D—F) IF co-staining of a first trimester placental tissue section showing p27 (D), p21 (E) and p16 (F) (magenta) and KRT7 (green) expression in EVTs. (G) IF co-staining of a first trimester placental tissue section showing HLA-G (magenta) and Cyclin A (green) expression of pCCTs and dCCTs. (H) IF co-staining of first trimester placental tissue showing Cyclin E (magenta) and p57 (green) expression of vCTBs and CCTs. (I) IF co-staining of first trimester decidual tissue showing Cyclin E (magenta) and p57 (green) expression of EVTs. Inset shows HLA-G (green) and DAPI (blue) staining of the same area. Digitally zoomed insets display a split-channel-depiction of the boxed areas. (blue) was used to visualize nuclei. (TIF) [file pgen.1007698.s002.tif]

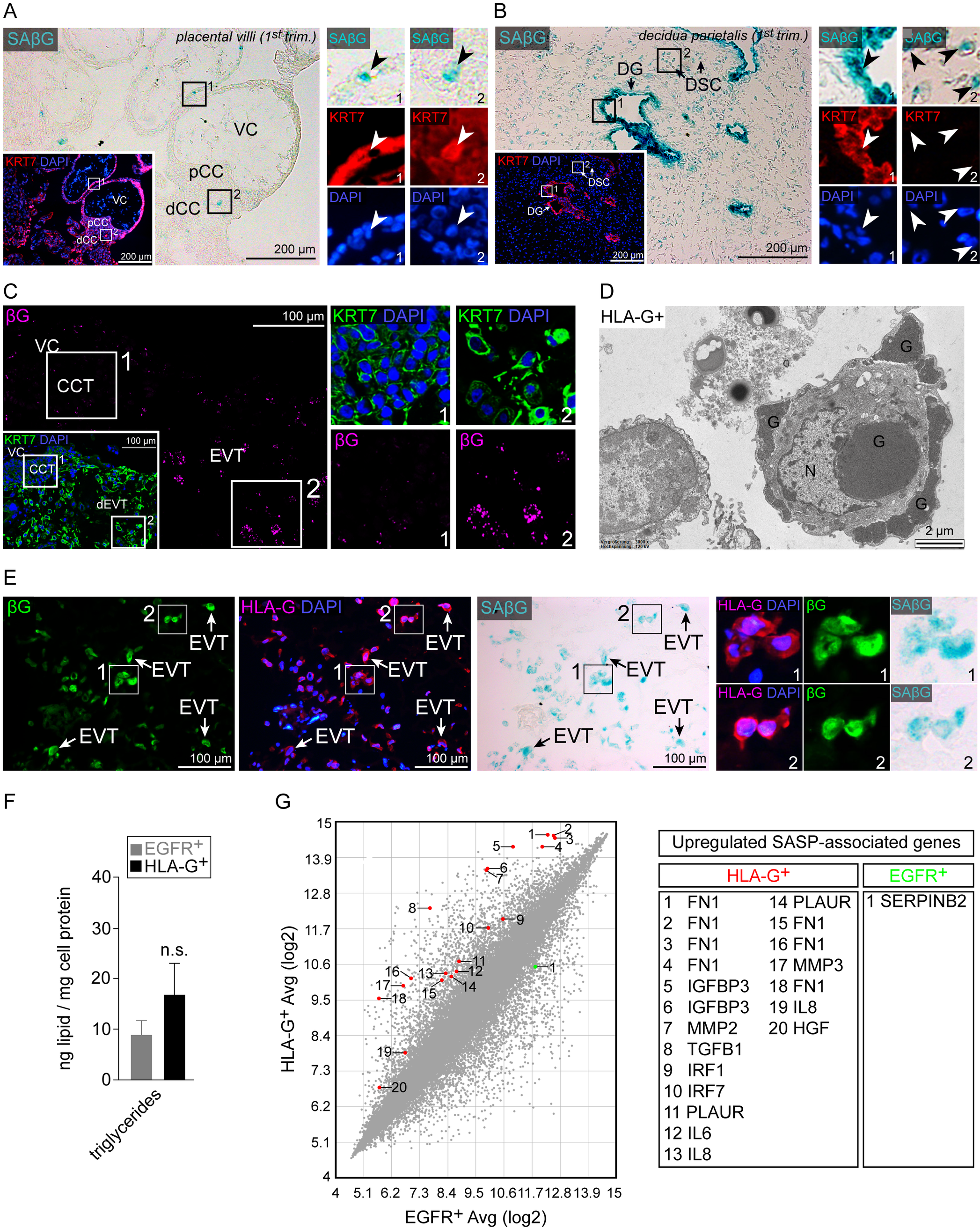

Supplement: S3 Fig — (A) Cryo-section of first trimester placental tissue showing co-stained SAβG activity (blue, large image) and Keratin7 (KRT7) (magenta, insert). Zoomed insets on the right show image details of the boxed areas, arrowheads indicate SAβG and KRT7 (red) positive trophoblast cells. (B) Cryo-section of first trimester decidua basalis tissue showing co-stained SAβG activity (blue, large image) and Keratin7 (KRT7) (magenta, insert). Zoomed insets on the right show image details of the boxed areas, arrowheads indicate SAβG and/or KRT7 (red) in decidual gland cells (left panel) and decidual stromal cells (right panel). (C) IF co-staining of first trimester placental tissue showing beta-galactosidase (βG, magenta, large image) and HLA-G (green, insert) co-staining. Zoomed insets on bottom show image details of the boxed area. (n = 3) (D) Representative electron microscopy image showing MACS-sorted, HLA-G+ primary human trophoblasts. (E) Section of first trimester cryo-embedded decidua basalis tissue (n = 3) showing SAβG activity (blue), HLA-G (magenta) and βG (green) co-staining. (F) Gas chromatography assistested analysis of triglyceride contents in isolated EGFR+ and HLA-G+ trophoblasts (n = 3). (G) Scatter blot (left panel) and table indicating significantly regulated SASP-associated genes in isolated EGFR+ and HLA-G+ trophoblasts. DAPI (blue) was used to visualize nuclei. (TIF) [file pgen.1007698.s003.tif]

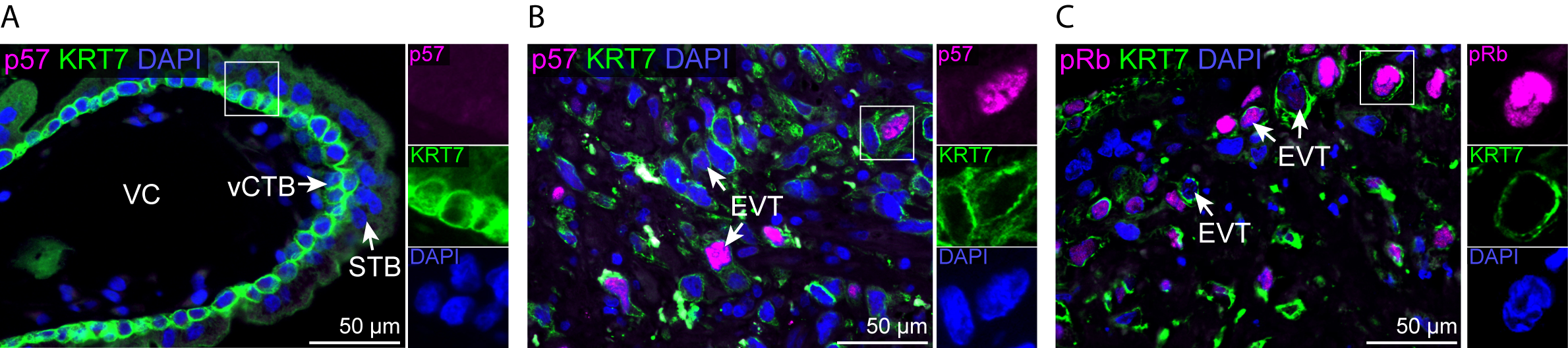

Supplement: S4 Fig — (A-B) IF co-staining of first trimester CHM placental tissues showing p57 (magenta) and KRT7 expression (green) in villous trophoblasts (A) and decidual EVTs (B). (C) Representative IF co-staining showing pRB (magenta) and KRT7 (green) expression in CHM-EVTs. DAPI (blue) was used to visualize nuclei. Digitally zoomed insets display a split-channel-depiction of the boxed area. (TIF) [file pgen.1007698.s004.tif]

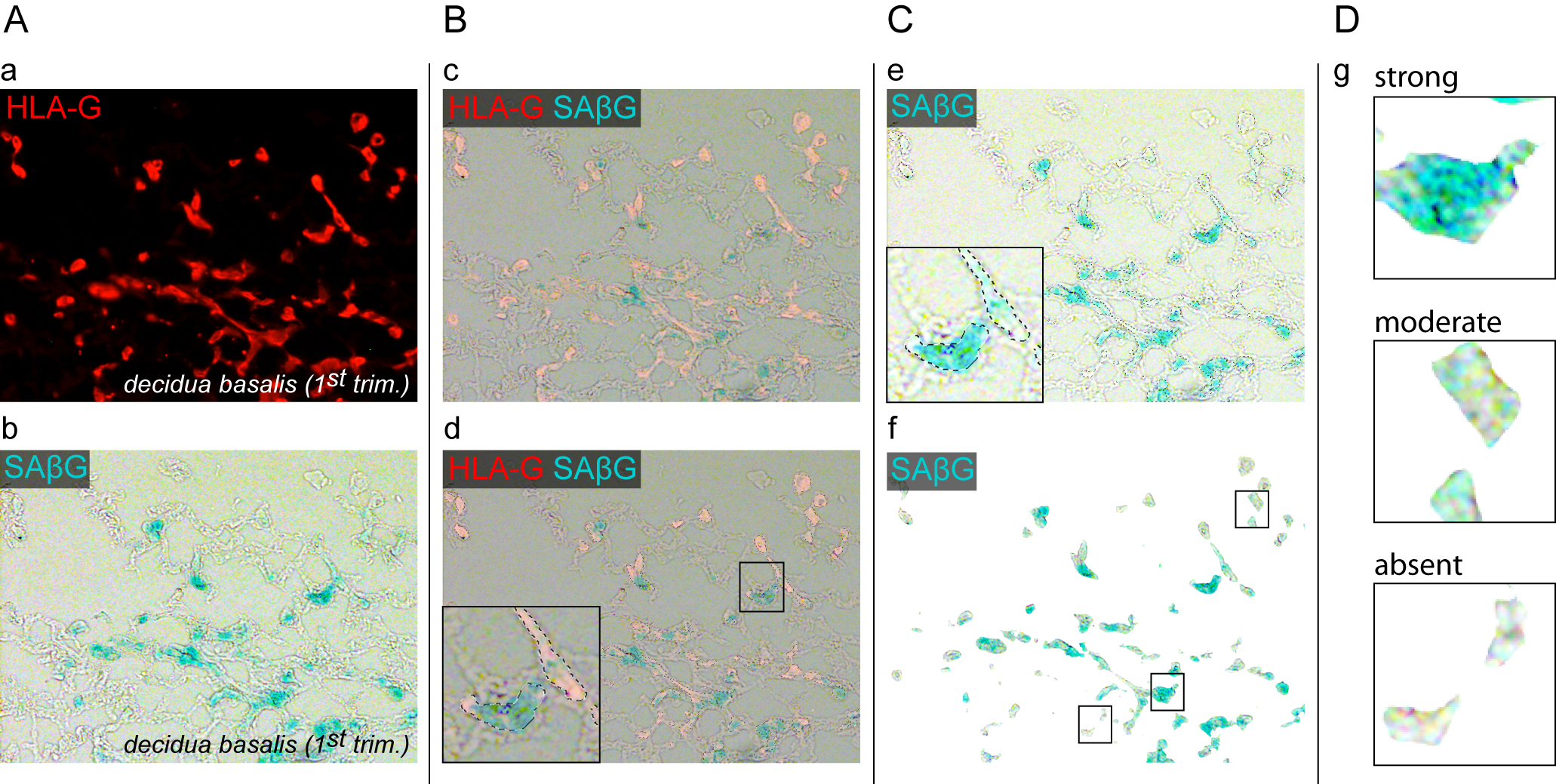

Supplement: S5 Fig — (A) Cryo-sectioned decidua basalis tissues were treated with SAβG activity assay and counterstained with an antibody against HLA-G (a-b). (B) Subsequently, fluorescence and bright field images were overlaid in Photoshop (c) and HLA-G+ areas were selected using the Magic Wand tool (d). (C) The bright field channel was then isolated (e) and pre-selected HLA-G+ areas were cropped (f). (C) Finally, SAβG signals were quantified as indicated in (g). (TIF) [file pgen.1007698.s005.tif]
