# Supplementary material for: Genome amplification and cellular senescence are hallmarks of human placenta development
Source: PLoS Genet. 2018 Oct 12;14(10):e1007698. doi: 10.1371/journal.pgen.1007698 (PMC6200260; doi:10.1371/journal.pgen.1007698)
Supplement: S1 Table — (PDF) [file pgen.1007698.s006.pdf]

**Table S1 Primary and secondary antibodies.**

| Antibody | Cat.# | Dilution | Company | Species | Application |
|---|---|---|---|---|---|
| Aurora B | Ab3609 | 1:100 | Abcam | Mouse | IF-P |
| Cathepsin A | MAB1049-S | 1:200 | R&D | Mouse | IF-P |
| Cyclin A | MS-1061-S | 1:50 | Neomarkers | Mouse | IF-P |
| Cyclin B | 610220 | 1:100 | BD Biosciences | Mouse | IF-P |
| Cyclin E | sc-247 | 1:100 | Santa Cruz | Mouse | IF-P |
| Cytokeratin7 | M7018 | 1:200 | DAKO | Mouse | IF-P |
| Cytokeratin (488 conjugated) | A-31556 | 1:100 | Eubio | Mouse | IF-P |
| EGFR | 4267 | 1:50 | Cell Signaling | Rabbit | IF-P |
| β-galactosidase | sc-20161 | 1:100 | Santa Cruz | Rabbit | IF-P |
| HLA-G | NB500-302 | 1:200<br>1:500 | Novus Biologicals | Mouse | IF-P<br>WB |
| IL-6 | NCL-L-IL6 | 1:50 | Leica Biosystems | Mouse | IF-P |
| Ki67 | M7240 | 1:100 | DAKO | Rabbit | IF-P |
| p16 | Sc-56330 | 1:150 | Santa Cruz | Mouse | IF-P |
| p21 | 2947 | 1:800 | Cell Signaling | Rabbit | IF-P |
| P27 | 3686 | 1:800 | Cell Signaling | Rabbit | IF-P |
| p57 | sc-1040 | 1:100 | Santa Cruz | Rabbit | IF-P |
| pH2A.X (Ser139) | 9718 | 1:100 | Cell Signaling | Rabbit | IF-P |
| PCNA | 2586 | 1:3000 | Cell Signaling | Mouse | IF-P |
| Vimentin | Ab16700 | 1:200 | Abcam | Rabbit | IF-P |
| phospho-H3 | 9701S | 1:200 | Cell Signaling | Rabbit | IF-P |
| Phospho-Rb | 8516 | 1:100 | Cell Signaling | Rabbit | IF-P |
| GAPDH | 14C10 | 1:5000 | Cell Signaling | Rabbit | WB |
| Anti-mouse 488 | A11017 | 1:1000 | Invitrogen | Goat | IF-P |
| Anti-rabbit 488 | A11070 | 1:1000 | Invitrogen | Goat | IF-P |
| Anti-mouse 568 | A11019 | 1:1000 | Invitrogen | Goat | IF-P |
| Anti-rabbit 421 | 406410 | 1:100 | BioLegend | Donkey | |
| Anti-rabbit 568 | A11011 | 1:1000 | Invitrogen | Goat | IF-P |

| | | | | | |
|---|---|---|---|---|---|
| HLA-G-PE | 1P-292-C100 | 5 µg/ml | Exbio | Mouse | MACS |
| EGFR-PE | sc-101 | 1µg/$10^6$ cells | Santa Cruz | Mouse | MACS |
| HLA-G-PE | 1P-292-C100 | 1:100 | Exbio | Mouse | FC |
| EGFR-488 | sc-101 | 1µg/$10^6$ cells | Santa Cruz | Mouse | FC |
| PE Mouse IgG1 | 550617 | 1:10 | BD | Mouse | FC |
| PE Mouse IgG2b | 555058 | 1:10 | BD | Mouse | FC |

IF-P, Immunofluorescence of paraffin sections; FC, Flow cytometry; MACS, Magnetic-activated cell sorting; WB, Western blot
